# Multi-locus genome-wide association studies reveal novel alleles for flowering time under vernalisation and extended photoperiod in a barley MAGIC population

**DOI:** 10.1007/s00122-022-04169-x

**Published:** 2022-07-25

**Authors:** Viet Hoang Dang, Camilla Beate Hill, Xiao-Qi Zhang, Tefera Tolera Angessa, Lee-Anne McFawn, Chengdao Li

**Affiliations:** 1 Western Crop Genetics Alliance, Agricultural Sciences, College of Science, Health, Engineering and Education, Murdoch University, Murdoch, Perth, WA Australia; 2 Department of Primary Industries and Regional Development, Perth, WA Australia

## Abstract

**Key message:**

Key genes controlling flowering and interactions of different photoperiod alleles with various environments were identified in a barley MAGIC population. A new candidate gene for vernalisation requirements was also detected.

**Abstract:**

Optimal flowering time has a major impact on grain yield in crop species, including the globally important temperate cereal crop barley (*Hordeum vulgare* L.). Understanding the genetics of flowering is a key avenue to enhancing yield potential. Although bi-parental populations were used intensively to map genes controlling flowering, their lack of genetic diversity requires additional work to obtain desired gene combinations in the selected lines, especially when the two parental cultivars did not carry the genes. Multi-parent mapping populations, which use a combination of four or eight parental cultivars, have higher genetic and phenotypic diversity and can provide novel genetic combinations that cannot be achieved using bi-parental populations. This study uses a Multi-parent advanced generation intercross (MAGIC) population from four commercial barley cultivars to identify genes controlling flowering time in different environmental conditions. Genome-wide association studies (GWAS) were performed using 5,112 high-quality markers from Diversity Arrays Technology sequencing (DArT-seq), and Kompetitive allele-specific polymerase chain reaction (KASP) genetic markers were developed. Phenotypic data were collected from fifteen different field trials for three consecutive years. Planting was conducted at various sowing times, and plants were grown with/without additional vernalisation and extended photoperiod treatments. This study detected fourteen stable regions associated with flowering time across multiple environments. GWAS combined with pangenome data highlighted the role of *CEN* gene in flowering and enabled the prediction of different *CEN* alleles from parental lines. As the founder lines of the multi-parental population are elite germplasm, the favourable alleles identified in this study are directly relevant to breeding, increasing the efficiency of subsequent breeding strategies and offering better grain yield and adaptation to growing conditions.

**Supplementary Information:**

The online version contains supplementary material available at 10.1007/s00122-022-04169-x.

## Introduction

Over the last 40 years, the development of new barley cultivars combined with improved management practices has boosted Australia’s grain yield production rate to over 40 kg/ha each year (Anderson et al. 2005). With such improvements, barley grain yield in Australia had increased from 0.98 tons/ha in 1961 to 2.40 tons/ha in 2014. Despite these substantial gains, Australian barley yield still lags behind European countries such as Germany, which successfully increased their barley grain yield from 2 tons/ha in the early nineteenth century to a current level of more than 7 tons/ha (Friedt et al. 2011).

Optimal flowering time plays a significant role in grain yield improvement and has been targeted in many barley breeding programs. A later flowering time might provide plants with more extended growing periods, increasing the time to accumulate nutrients, and may result in a higher grain yield. It can also help plants to avoid frost damage, since the critical period of high frost sensitivity is linked to spikelet survival and the number of grains per spike ranges from awn primordia development to heading (Alqudah and Schnurbusch 2014; Liu et al. 2020). However, later flowering can also increase the severeness of terminal heat and drought stress, which negatively impacts fertility and grain yield (Rana et al. 2017; Samarah 2005; Savin and Nicolas 1999). Moreover, water availability is crucial in maintaining barley yields, since water stress can significantly reduce the number of grain and grain weight, especially in many low rainfall growing regions (Samarah 2005; Savin and Nicolas 1999). Therefore, extending and matching the critical flowering period with optimal environmental conditions can help boost barley yield potential.

Many key genes and signalling pathways affecting flowering time as well as grain yield have been identified, and their interactions with the environment evaluated, particularly in model plants such as Arabidopsis and *Oryza sativa* (Colasanti and Coneva 2009; Fjellheim et al. 2014). In barley, the most significant pathways that affect flowering time are photoperiod (duration of exposure to light), vernalisation (prolonged exposure to low temperature) and earliness per se (influence the time of flowering independently of environmental stimuli) (Andres and Coupland 2012; Cockram et al. 2007).

In recent years, several studies have been conducted to detect Quantitative trait loci (QTLs) associated with flowering time in barley. Coventry et al. (2003) reported that *Ppd-H1* and *Earliness *per se* 2* (*eps2*) loci are related to grain size and grain weight performance in an Australian barley population. Cuesta-Marcos et al. (2009) suggested that there are five main QTLs, including *Photoperiod 2* (*Ppd-H2*) and *eps2* (or *Early maturity 6*, *Eam6*), that directly affect barley heading date and grain yield under autumn sowing conditions in Spain. Furthermore, Borràs-Gelonch et al. (2011) also reported that *Ppd-H1* and *Eam6* significantly affected flowering time in the Steptoe x Morex population. Alqudah et al. (2014) divided a mapping population based on photoperiodic response levels and suggested that Ppd-H1, Ppd-H2 and Constans 1 (CO1) genes play a role major role in early heading under long-day conditions in barley. Another study conducted by Maurer et al. (2015) used nested association mapping (NAM) to detect eight major QTLs controlling flowering time in barley, with the major QTL effect corresponding to the photoperiod response gene *Ppd-H1*.

In the vernalisation pathway, the core gene *Vernalisation 1* (*Vrn-H1*), located on the long arm of chromosome 5H, was reported to promote flowering following prolonged cold temperature (Cockram et al. 2007). The up-regulation of the *Vrn-H1* gene lead to the repression of the *Vernalisation 2* (*Vrn-H2*) and consequently promoting the *Flowering locus T 1* (*FT1*) gene (Sasani et al. 2009). A previous study reported that a deletion in the regulatory site of *Vrn-H1* combined with the deletion of *Vrn-H2* leads to a spring growth habit (Rollins et al. 2013). In spring barley, the combination of overexpression of *Constans 2* (*CO2*) and a deletion in the *Vrn-H2* region leads to floral transition independent on *Ppd-H1* induction. In winter barley, the overexpression of *CO1* or *CO2* leads to the upregulation of *Vrn-H2*, reducing the expression of *FT1,* which results in a delay in flowering.

Although many genes and QTLs associated with flowering have been reported, little information is available on functional alleles underlying these QTLs present in adapted domesticated lines. Crop populations derived from crosses of adapted domesticated cultivars enable the genetic dissection of complex traits while also providing allelic information of agronomically relevant cultivars, increasing the efficiency of subsequent breeding strategies. Multi-parent populations play a central role, as they combine many beneficial properties of genetic mapping populations by recombining the genomes of multiple parental varieties. In addition, multi-parent populations offer better detection power, higher resolution for mapping quantitative trait loci and higher genetic diversity than traditional bi-parental populations (Huang et al. 2015). These properties are important for designing allele-specific markers for marker-assisted breeding (MAS) (Gupta et al. 2014; Myles et al. 2009). In recent years, multi-parent populations were successfully used for high-resolution mapping of awn length in barley (Liller et al. 2017) and phenology traits in wheat (Camargo et al. 2016). Obsa et al. (2016) successfully identified eight maturity QTLs associated with phenology genes and eighteen QTLs responsible for adaptive traits in barley using DHs derived from crosses of three parents Keel, Sloop and Galaxy. Hemshrot et al. (2019) reported using a multi-parental population generated from crosses between 92 donor parents and Rasmusson to capture more genetic diversity and identify new haplotypes for the *Ppd-H1* gene.

In this study, we used a multi-parent approach to explore the genetic diversity in elite germplasm directly relevant to breeding. A Multi-parent advanced generation intercross (MAGIC) population was constructed to take advantage of four commercial barley cultivars developed in different germplasm programs: Compass (Victoria, Australia), GrangeR (Europe), Lockyer (Western Australia) and La Trobe (South Australia). A total of 184 recombinant inbred lines (RILs) with maximum diversity in plant growth and phenology development were selected from the MAGIC population and sequenced using Diversity Array Technology (DArT) to obtain a total of 10,066 markers for GWAS. To provide additional coverage for the most important phenology and semi-dwarf genes as published previously in Dang et al. (2020); Hill et al. (2019a, b, 2021) a total of 121 KASP markers, specifically targeting 66 key phenology genes, were also developed. Multi-locus GWAS methods were used to overcome the highly structured population and high linkage disequilibrium (LD), provide an in-depth overview of the contribution of major phenology genes to phenology development, and identify novel genes associated with flowering time in barley.

The purpose of this study was to identify loci associated with flowering time under extended and natural light, as well as under vernalised and non-vernalised conditions with high resolution using a MAGIC population of 184 recombinant inbred lines (RILs). Beneficial parental alleles from these founders of the MAGIC population were identified for fine-tuning of flowering time in targeted growing conditions. Trials for three consecutive years, from 2017 to 2019, were set up in Perth, Western Australia, to identify stable candidate genes controlling flowering time in various environmental conditions. In addition, two more trials were conducted in Corrigin and Esperance, Western Australia, in 2018. Different sowing times (ranging from April to August) were used to investigate the association of candidate genes with day-length and temperature on flowering time, which can be used as references for other growing sites with similar conditions across Australia. Vernalisation treatment and extended photoperiod conditions were used to identify genes responsible for controlling flowering under low temperature and prolonged day length.

## Material and methods

### Multi-parental population

The MAGIC population were constructed using four commercial barley cultivars Compass, GrangeR, Lockyer, La Trobe and previously described in Dang et al. (2020). The parental cultivars were chosen based on their outstanding performance and exhibit high grain yield potential and stability in various Australian production zones. Furthermore, these varieties represent germplasm pools from four major breeding programs, which are based in different locations across Australia and Europe, and were shown to contain high genetic variability in phenology genes (Hill et al. 2019a, b).

These founders were crossed pairwise (Compass × GrangeR and Lockyer × La Trobe) to create two F_1_ populations. The F_1_ lines were then inter-crossed to generate 580 four-way (Compass/GrangeR × Lockyer/La Trobe) F_2_ and ~ 2000 F_3_ plants. The F_3_ population was advanced to the F_6_ generation via single-seed descent (Brim 1966).

One hundred eighty-four RILs were selected from the 2,000 F6 plants based on their differences in flowering time, plant height and grain yield as material for genotyping-by-sequencing by DArTseq and KASP assays. Moreover, these 184 RILs were also selected to have different genotypic backgrounds of the *sdw1* gene, including the *sdw1.d* and *sdw1.Lockyer* alleles, and the *ari-e* gene Dang et al. (2020).

### Phenotypic data collection

Phenotypic data were collected from 15 trials across Western Australia from 2017 to 2019 (Table S1). To investigate the seasonal impact, three trials were conducted in Perth, Western Australia, in three consecutive years from 2017 to 2019. The impact of different locations on flowering time was also assessed in two trials conducted in Corrigin and Esperance in 2018. Ten trials with five different sowing dates spanned from April to August were conducted in Perth to investigate the impact of different sowing times and vernalisation on flowering. Each sowing date included two trials: non-vernalised and vernalised. In the vernalisation experiments, seedlings were subjected to cold temperature at 4 °C for 4–6 weeks before transplanting to the field for phenology scoring. One extended photoperiod trial was also conducted in Perth with an 18-h photoperiod.

Optimum management practices were applied for each trial, including weeding, disease control and fertilising based on local environmental conditions. Although flowering time in barley has been previously characterised by the anthesis stage in the Zadoks’ decimal scale (Z60-69, Zadok et al. 1974), a recent study by Alqudah and Schnurbusch (2017) shows that in spring barley, anthesis or fertilisation happens around the awn tipping stage. Therefore, in this study, flowering time was as approximated using the awn appearance stage (Z49) as described in Dang et al. (2020); Hill et al. (2021).

### Genotyping-by-sequencing by DArTseq and KASP assays

DNA samples from 184 selected RILs and four parental cultivars were sequenced using DArT to obtain 10,066 high-quality single-nucleotide polymorphism (SNP) markers for association analyses. DArT-Seq genotyping by sequencing (GBS) was performed using the DArT-Seq platform (DArT PL, Canberra, NSW, Australia) as described on the company website (https://www.diversityarrays.com). Briefly, 100 μl of genetic material at 50 ng/μL was sent to DArT PL, and GBS was performed using a combination of complexity reduction followed by sequencing on a HiSeq Illumina platform (Illumina Inc., San Diego, CA, USA) as described by Akbari et al. (2006). Marker sequences were aligned against the Morex barley genome assembly (Mascher et al. 2017). The genetic position of each marker was determined based on the Morex physical reference assembly.

One hundred twenty-one KASP markers distributed over 66 phenology-related genes were developed to provide better coverage of different phenology gene haplotypes based on genetic information published for the four parental lines by Hill et al. (2019a, b, 2021). These genes were previously reported to affect phenology, grain yield and plant height under different environmental conditions in Western Australia.

Genetic markers for KASP assays were designed based on the technology from LGC genomics (https://www.lgcgroup.com). KASP assays were conducted using 5 µl reactions containing 50 ng of high-quality DNA template extracted from 3-week-old leaves, with annealing temperature reduced from 61 to 57 °C in the first ten cycles. The results were analysed using QuantStudio Real-Time PCR v1.3 software (Applied Biosystems, Australia).

### Genetic variant filtering and imputation

The genotypic dataset was generated for 188 plant lines (184 selected RILs and four parental cultivars), containing 10,066 SNP markers from DArT-seq, 121 KASP-based SNP markers covering major phenology genes, and three allele-specific markers for the semi-dwarf genes *sdw1* and *ari-e* (Table S2). The data were converted to Variant Call Format (VCF v4.0) and filtered to remove variants with minor allele frequency (MAF) lower than 5% and missingness higher than 20% using PLINK 1.9 (Purcell et al. 2007). Genetic variants with heterozygous rates over 25% or showing no polymorphism among the four parental lines were also removed. The filtered marker set was imputed using BEAGLE 5.0 (Browning et al. 2018) and pruned using PLINK 1.9 (Purcell et al. 2007).

### Linkage disequilibrium and Population structure analysis

Genome-wide LD analysis was performed using PLINK 1.9 (Purcell et al. 2007) with 5,112 filtered and imputed genetic markers for 184 RILs and four parental cultivars. LD was estimated using squared allele frequency correlations (*r*^2^) between the intra-chromosomal pairs of loci (Weir 1996). The loci were considered to be in significant LD when *P* < 0.001.

To investigate LD decay in the population, significant inter-chromosome *r*^2^ values within each 100-kb bin were plotted against the physical distance (kb) between markers. Curves were fitted by a second-degree LOESS function using R 3.6.1 (R Core Team 2013). Since the population is highly structured and not all genetic markers are informative, the marker set was further pruned based on the r^2^ threshold of 0.8 using PLINK 1.9 (Purcell et al. 2007). The pruned dataset containing 2,207 genetic markers was used for population structure analysis and phylogenetic tree construction.

Population structure analysis was performed using Admixture 1.3.0 with different clusters (*K*) from 1 to 20 with 100 replications, and suitable *K* was selected based on cross-validation results (Alexander et al. 2009). The result was then collected, analysed and visualised using the R package “Pophelper 2.3.1” (Francis 2017) and CLUMPP v1.1.2 (Jakobsson and Rosenberg 2007). A phylogenetic tree was constructed based on the distance matrix calculated as 1 - IBS (identity by state) similarity by TASSEL 5.0 (Bradbury et al. 2007) and visualised with FigTree 1.4 (Rambaut 2012).

### Genome-wide association analysis

To investigate the vernalisation response, variation in flowering time between vernalised and non-vernalised were calculated using the following formula:

$$Z49_{{{\text{Variation}}}} = Z49 _{{{\text{Vernalised}}}} - Z49_{{\text{Non - vernalised}}}$$

where Z49_Vernalised_ and Z49_Non-vernalised_ are defined as date to awn appearance from sowing (flowering time) of a line grown under vernalisation treatment or under natural light conditions.

Genome-wide association analysis was performed using a total of 5,112 genetic variants present in 188 individual lines with different methods provided by the R packages “mrMLM v4.0.2”. These methods include multi-locus random-SNP-effect mixed linear model (mrMLM); fast multiple-locus random-SNP-effect mixed linear model (FASTmrMLM); fast multi-locus random-SNP-effect efficient mixed-model association (FASTmrEMMA); least angle regression with empirical Bayes (pLARmEB); integrative sure independence screening expectation maximisation Bayesian least absolute shrinkage and selection operator model (ISIS EM-BLASSO) and Kruskal–Wallis test with empirical Bayes under polygenic background control (pKWmEB) (Zhang et al. 2020). The Q + K model was used, with the population structure matrix Q calculated by Admixture and the kinship matrix *K* calculated using the “mrMLM” packages. The default parameters were used, with the significance of Logarithm of Odds (LOD) of 3 to determine significant Quantitative Trait Nucleotides (QTNs) associated with flowering time. A search radius of 20 kb for candidate genes was used in mrMLM and FASTmrEMMA, and 50 potential association loci on each chromosome for pLARmEB (Zhang et al. 2020).

The linkage of markers identified by GWAS was visualised with Haploview (Barrett et al. 2004). Significant QTNs from GWAS results from multiple trials were collected and visualised using Circos 0.69 (Krzywinski et al. 2009).

### Pangenome sequence analysis

The pan-genome data of the genome sequences from 20 different barley accessions were obtained from the Leibniz Institute of Plant Genetics and Crop Plant Research (https://barley-pangenome.ipk-gatersleben.de) (Jayakodi et al. 2020). The reference sequence of the *CEN* gene was obtained from the Morex genome sequence and blasted against the pan-genome dataset to identify the copy number as well as the physical position of the *CEN* gene on each of the accession sequences using “blastn” (Altschul et al. 1990). The *CEN* gene sequence from each pan-genome assembly was extracted, and multiple alignments were performed using Muscle 3.8.31 (Edgar 2004) to obtain genetic variants.

The phylogenetic tree was constructed using TASSEL 5 (Bradbury et al. 2007) with the high-quality SNP data of the *CEN* gene were obtained from the previous studies of Hill et al. (2019a, b) and the SNP data derived from sequence alignment.

## Results

### Impact of vernalisation treatment and extended photoperiod on flowering time

**Fig. 1:**
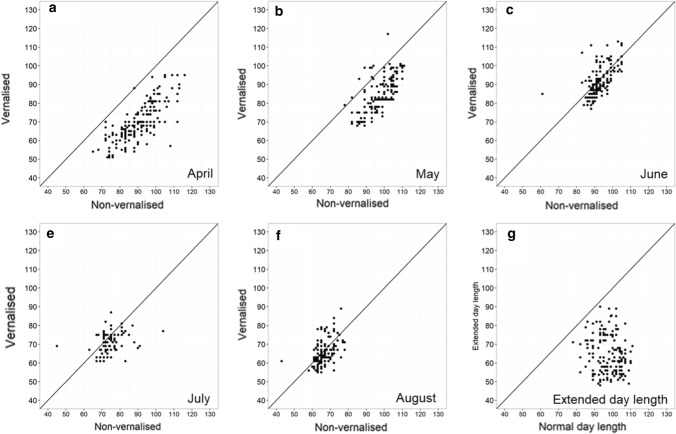
Scatter plot comparing the flowering time between vernalised, extended photoperiod and normal conditions. Each box represents a different trial conducted in Perth in 2019 with sowing time in **a** April, **b** May, **c** June, **d** July and **e** August; and comparing **f** extended and regular photoperiod. Dots depicted the date to awn appearance (Z49) of individual plants

Plants subjected to vernalisation treatment at 4 °C for 4–6 weeks showed earlier flowering in all trials compared to non-vernalised ones (Fig. 1a–e). The impact of vernalisation was more significant for earlier sowing times, with an average of 21 and 13.3 days earlier in flowering when sowed in April and May, respectively (Table S3). However, vernalisation treatment showed minimal impact on plants sown in July and August. The differences in flowering time between vernalised and non-vernalised plants were only 3.4 and 1.2 days in July and August, respectively (Table S3). Plants grown under extended day length conditions exhibited earlier flowering habits, with an average of 33.7 days earlier than plants grown under normal light conditions (Fig. 1f and Table S3).

### Population structure and LD analysis

Initial filtering with MAF and missingness resulted in the removal of 2,434 markers (23%) from the dataset. Further filtering of the imputed dataset suggested no major changes after the imputation process, with all markers passing the filtering threshold. The minor allele frequency of the dataset before and after filtering and imputing is shown in Fig. S1a, b. The filtered and imputed marker set were used for LD analysis. The LD analysis result suggested strong linkages between markers even at long distances of up to 10 Mb (long-distance LD) (Fig. S1c).

The phylogenetic tree constructed from the neighbour-joining tree results calculated by TASSEL 5.0 (Bradbury et al. 2007) showed that the RILs had different genetic combinations of the four parental cultivars, with groups highly related to a single parental cultivar as well as groups sharing genetic combinations of multiple cultivars (Fig. S2).

**Fig. 2:**
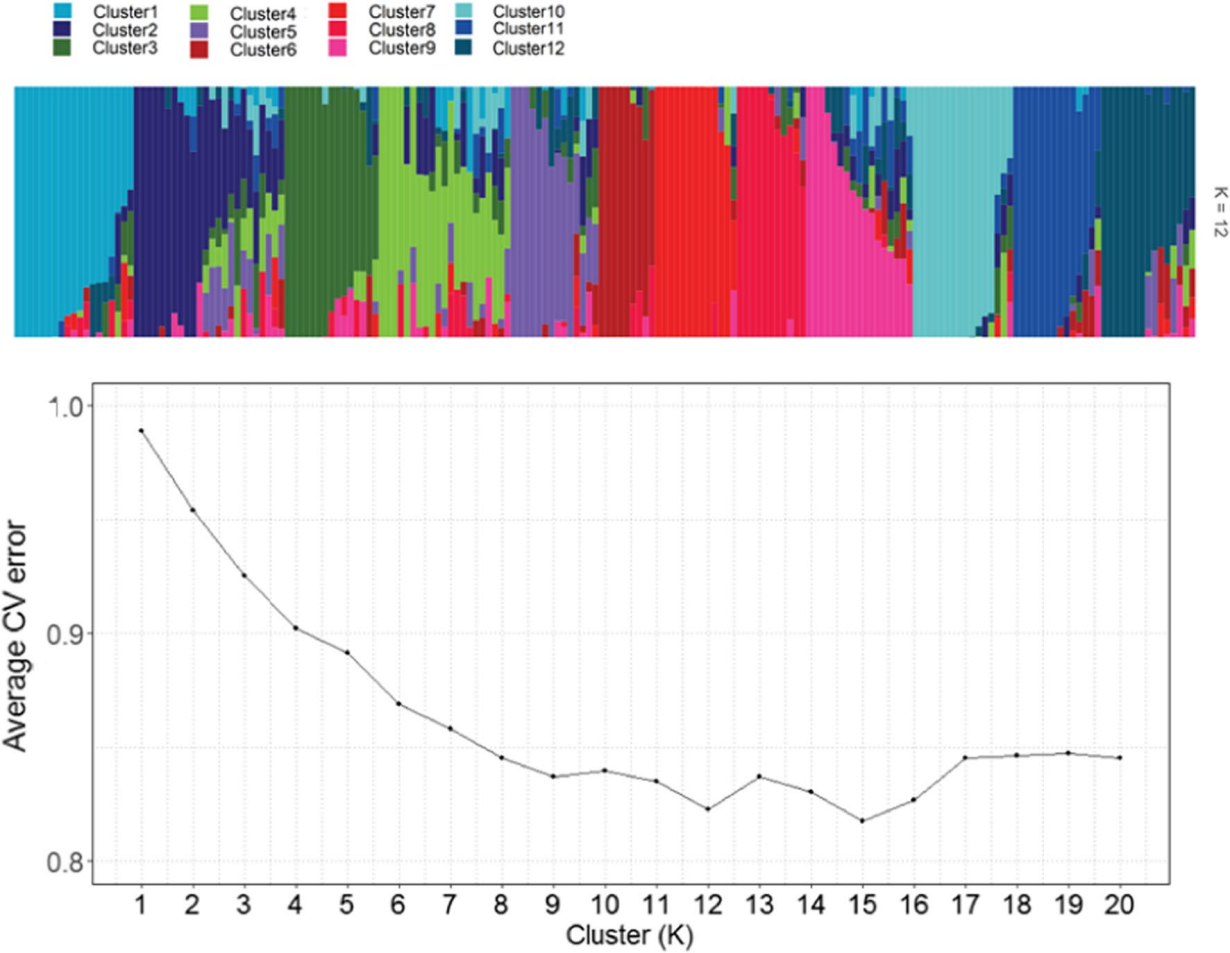
Population structure analysis result. **a** Ancestry plot of the population structure data from Admixture 1.3.0 12 clusters (*K* = 12) and **b** average cross-validation error of different K values from 1 to 20 from population structure analysis using Admixture 1.3.0

The cross-validation (CV) result from population structure analysis showed that the average CV error dropped significantly from 0.989 to 0.869 when *K* increased from 1 to 6. This value then decreased slightly to 0.822 at *K* of 12 before increase slightly and dropped to the lowest value at *K* of 15 (0.817). Therefore, the population analysis result of *K* = 12 was collected using CLUMPP (Jakobsson and Rosenberg 2007) and visualised using the R package “Pophelper” (Francis 2017) (Fig. 2). The Q matrix from population analysis result of *K* = 12 was also used for later GWAS.

### QTNs associated with flowering time

**Fig. 3:**
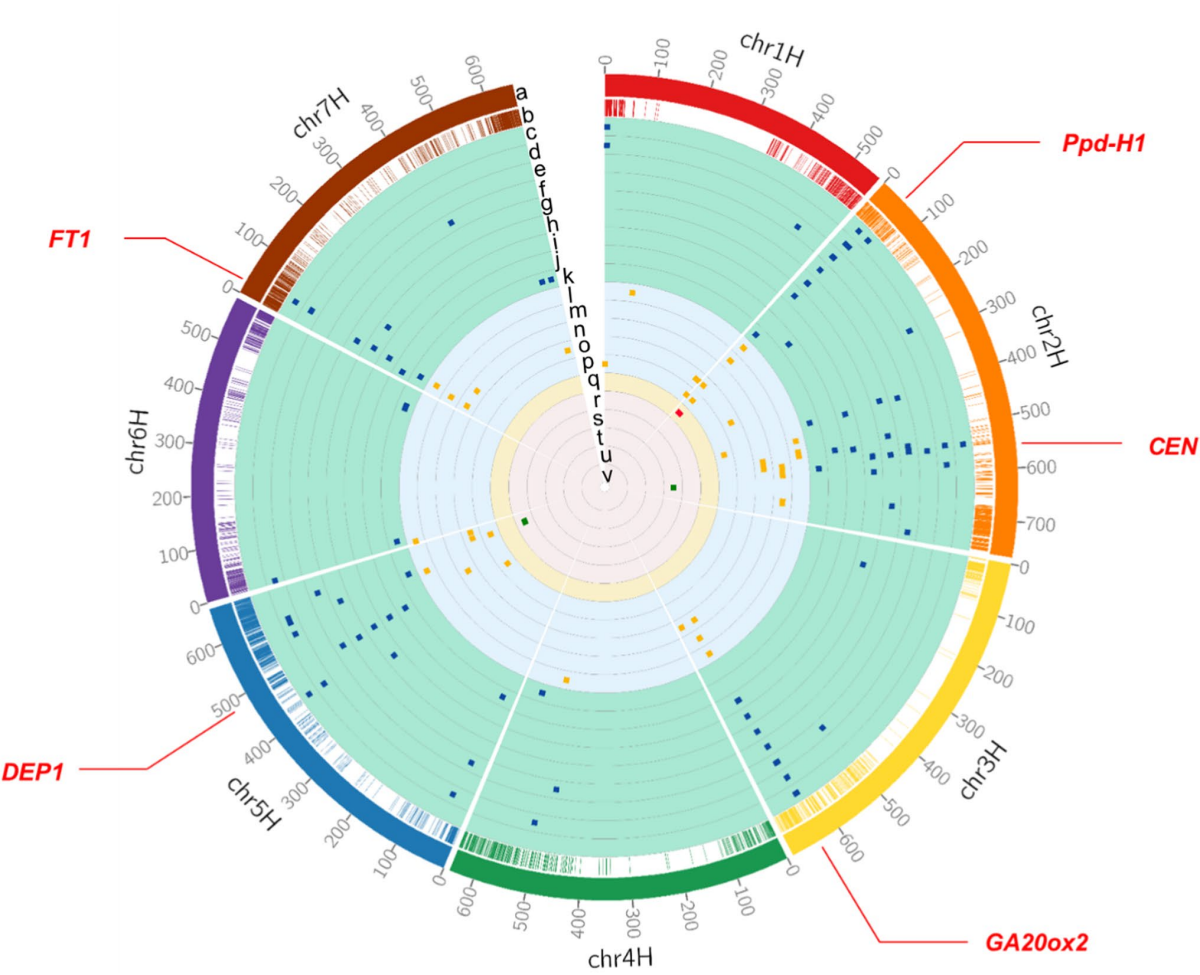
Genome-wide distribution of significant QTNs detected by GWAS. **a** The outermost ring with the scale represents the 7 barley chromosomes. **b** The colour lines in the inside ring represent the marker positions. The blue scatters represent the positions of significant QTNs in normal conditions, with each ring representing different trials conducted in **c** Corrigin 2018, **d** Esperance 2018, **e** Perth 2017, **f** Perth 2018, different sowing times in Perth in **g** April, **h** May, **i** June, **j** July, **k** August in 2019, respectively. The yellow scatters represent the positions of significant QTNs in vernalised trials, with each ring represent vernalised trials with different sowing times in Perth in **l** April, **m** May, **n** June, **o** July, **p** August in 2019, respectively. **q** The red scatters and yellow ring represent the positions of significant QTNs in extended photoperiod trial. The green scatters represent the positions of significant QTNs in vernalised/non-vernalised comparison, with each ring representing the vernalised/non-vernalised comparison for different sowing times in **r** April, **s** May, **t** June, **u** July, **v** August in 2019, respectively

GWAS detected 154 unique and significant QTNs associated with flowering time for different years, locations, and sowing times (Table S4). Multiple QTNs were linked to previously known regions such as D2H28394307_TC, D2H29454480_CT and D2H31462699_TG (located in the *Ppd-H1* region). The *Ppd-H1* region were also strongly associated with flowering time in the extended photoperiod trial (Fig. 3q). Multiple QTNs, from D3H631680288_GC to D3H635379323_GC, are spanning *GA20ox2* region. It is worth noting that the In-Del KASP marker designed explicitly for a mutation in the *DEP1* gene (K5H482215793) was also associated with flowering in multiple trials. There are two significant QTNs on chromosome 7H, D7H37802896_AG and D7H40095600_TC, located in the region containing three genes previously reported to impact flowering: *MADS-box 25–2* (*MADS25-2*), *MADS-box 25–3* (*MADS25-3*) and *FT1* (Fig. 3).

A region spanned from genetic marker D2H498405412_AG to genetic marker D2H585450069_GA (approximately 100 Mb) showed a significant association with flowering in most of the trials. Linkage analysis suggested that these genetic markers are highly linked with LD scores ranging from 88 to 98 (Fig. S3).

Forty-two significant QTNs were detected in the vernalisation trials with different sowing times from April to August in 2019 in Perth (Fig. 3l–p and Table S4). The *Ppd-H1*, *GA20ox2* and *FT1* regions still showed significant association with flowering time following vernalisation treatment. The linkage region spanned from genetic marker D2H498405412_AG to D2H592062371_GA also detected by GWAS in multiple vernalisation trials (Fig. 3l–p).

Further analysis from GWAS results showed that 14 stable regions were detected. These regions contained significant QTNs associated with flowering time in at least two trials (Table 1). Besides major gene regions associated with flowering time in most of the trials (including *Ppd-H1*, *GA20ox2*, *DEP1*, *CEN*, *FT1*), there are several regions only showed association with flowering time in only 2 or 3 environments, such as *PFT1* gene at 5H:430,868,101 and 1H:4,653,106 (Table 1).

**Table 1:** Stable regions associated with flowering time in at least two different trials

Position/Inverval (bp)	Candidate gene	Haplotype 1	Haplotype 2	Trial
1H:4,653,106		Compass La Trobe	GrangeR Lockyer	Esperance 2018 Corrigin 2018
2H:28,394,307–31,462,699	*Ppd-H1*	Compass GrangeR	La Trobe Lockyer	Perth 2017 Perth 2018 Esperance 2018 Corrigin 2018 Perth April 2019 Perth May 2019 Perth August 2019 Perth April 2019 Vernalised Perth May 2019 Vernalised Perth 2019 Extended photoperiod
2H:37,222,451–38,635,094		Compass GrangeR	La Trobe Lockyer	Perth 2019 August Vernalised Perth 2019 Extended photoperiod
2H:271,995,988–271,999,272		Compass La Trobe Lockyer	GrangeR	Perth 2019 July Vernalised Esperance 2018
2H:402,416,491–453,687,559		Compass La Trobe Lockyer	GrangeR	Perth 2018 Perth April 2019 Perth June 2019 Perth August 2019 Perth April 2019 Vernalised Perth 2019 August Vernalised
2H:498,405,412–585,450,069	*CEN*	Compass La Trobe Lockyer	GrangeR	Perth 2017 Perth 2018 Corrigin 2018 Esperance 2018 Perth April 2019 Perth May 2019 Per June 2019 Perth July 2019 Perth August 2019 Perth April 2019 Vernalised Perth May 2019 Vernalised Perth 2019 June Vernalised
3H:631,680,288–635,379,323	*GA20ox2*	Compass La Trobe	GrangeR Lockyer	Perth 2017 Perth 2018 Corrigin 2018 Esperance 2018 Perth April 2019 Perth May 2019 Per June 2019 Perth April 2019 Vernalised Perth May 2019 Vernalised
5H:39,817,622–45,772,858		Compass GrangeR Lockyer	La Trobe	Perth 2018 Perth July 2019 Esperance 2018
5H:382,294,832–390,501,082		Compass GrangeR Lockyer	La Trobe	Perth May 2019 Perth 2019 August Vernalised
5H:430,868,101	*PFT1*	Compass	GrangeR La Trobe Lockyer	Esperance 2018 Corrigin 2018
5H:478,394,957–482,215,793	*DEP1*	Compass GrangeR Lockyer	La Trobe	Perth 2018 Perth April 2019 Perth May 2019 Per June 2019 Perth July 2019 Perth 2019 June Vernalised
5H:562,614,128–597,950,604	*Vrn-H1*	Compass La Trobe Lockyer	GrangeR	Esperance 2018 Perth April 2019 Perth August 2019 Perth April 2019 Vernalised Perth April 2019 Vernalised/Non-Vernalised
5H:628,711,504–651,511,097		Compass GrangeR Lockyer	La Trobe	Perth 2018 Perth 2019 July Vernalised
7H:37,802,896–40,095,600	*FT1*	Compass La Trobe	GrangeR Lockyer	Corrigin 2018 Esperance 2018 Perth May 2019 Per June 2019 Perth August 2019 Perth April 2019 Vernalised

*Ppd-H1* Photoperiod 1, *CEN* CENTRORADIALIS, *GA20ox2* Gibberellin 20 oxidase 2, *PFT1* Phytochrome and flowering time 1, *DEP1* Dense and erect panicle 1, *Vrn-1* Vernslisation 1, *FT1* Flowering locus T 1

### CENTRORADIALIS is the candidate gene for the high linkage region on chromosome 2H

A detailed investigation using the genetic markers spanned from D2H498405412_AG to D2H585450069_GA collected from GWAS showed that all the linked markers can be separated into two haplotypes, one inherited from cv. GrangeR and the other from cvs. Compass, La Trobe and Lockyer (Table S4).

**Fig. 4:**
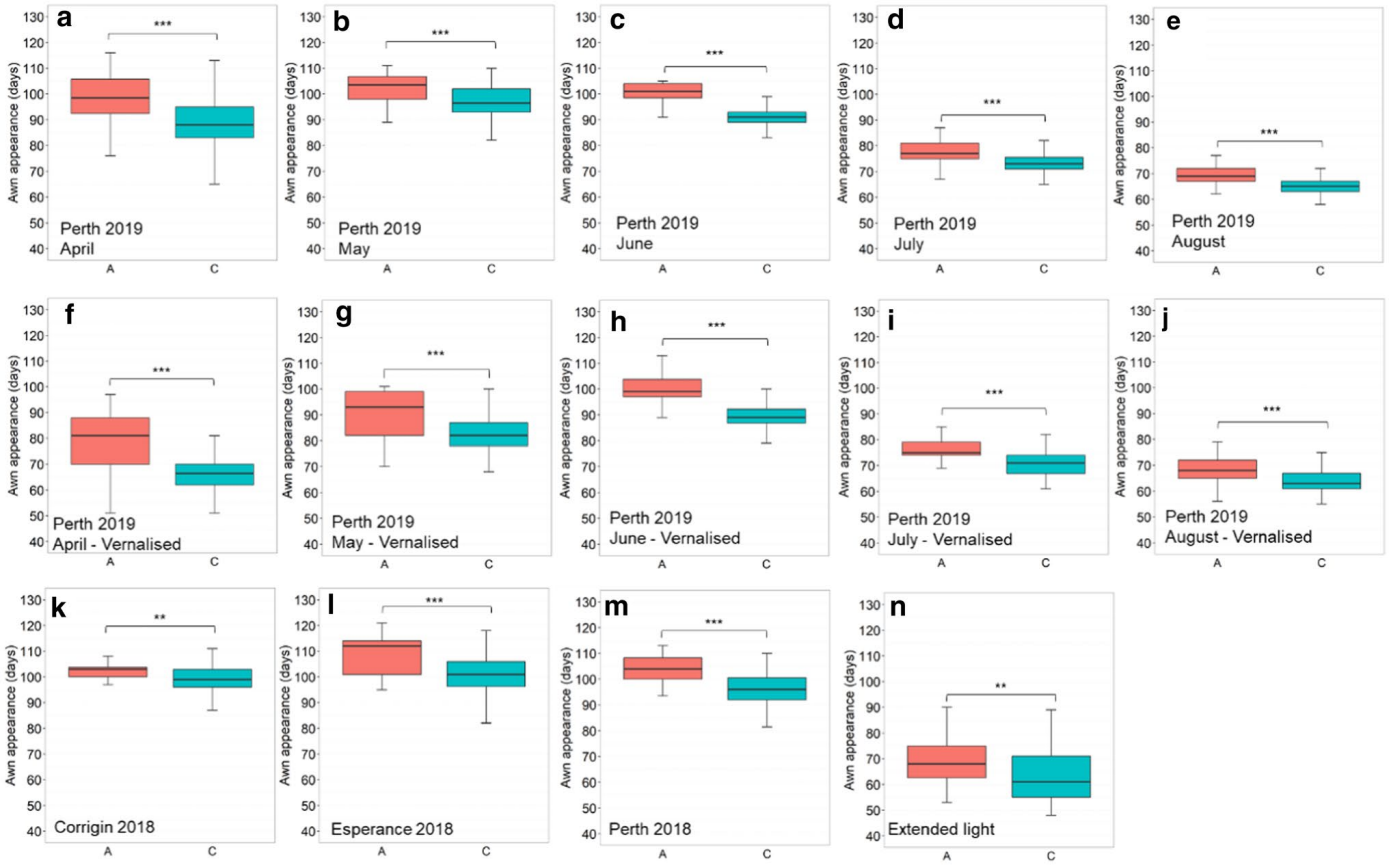
Boxplots for flowering time of different D2H519658782_AC QTN in different trials. **a**–**e** Non-vernalised trials with different sowing times from April to August in Perth 2019. **f**–**j**) Trials with vernalisation were sown from April to August in Perth 2019. **k**–**m** Trials at different locations in Corrigin, Esperance and Perth 2018. **n** Extended photoperiod trial. The population was divided into two groups according to allele types in each trial. The X-axis represents the two alleles, while the Y-axis corresponds to flowering time (Z49). Student’s t-test results shown for comparison: **p-value < 0.01, ***p-value < 0.001

Analysis of the D2H519658782_AC QTN located near the *CEN* region (523 Mb on chromosome 2H) suggested that the GrangeR genotype (A genotype) showed a significant delay in flowering time in all trials (Fig. 4). The A genotype exhibited up to 12.2 days delay in flowering compared to the C genotype (Table S5).

**Fig. 5:**
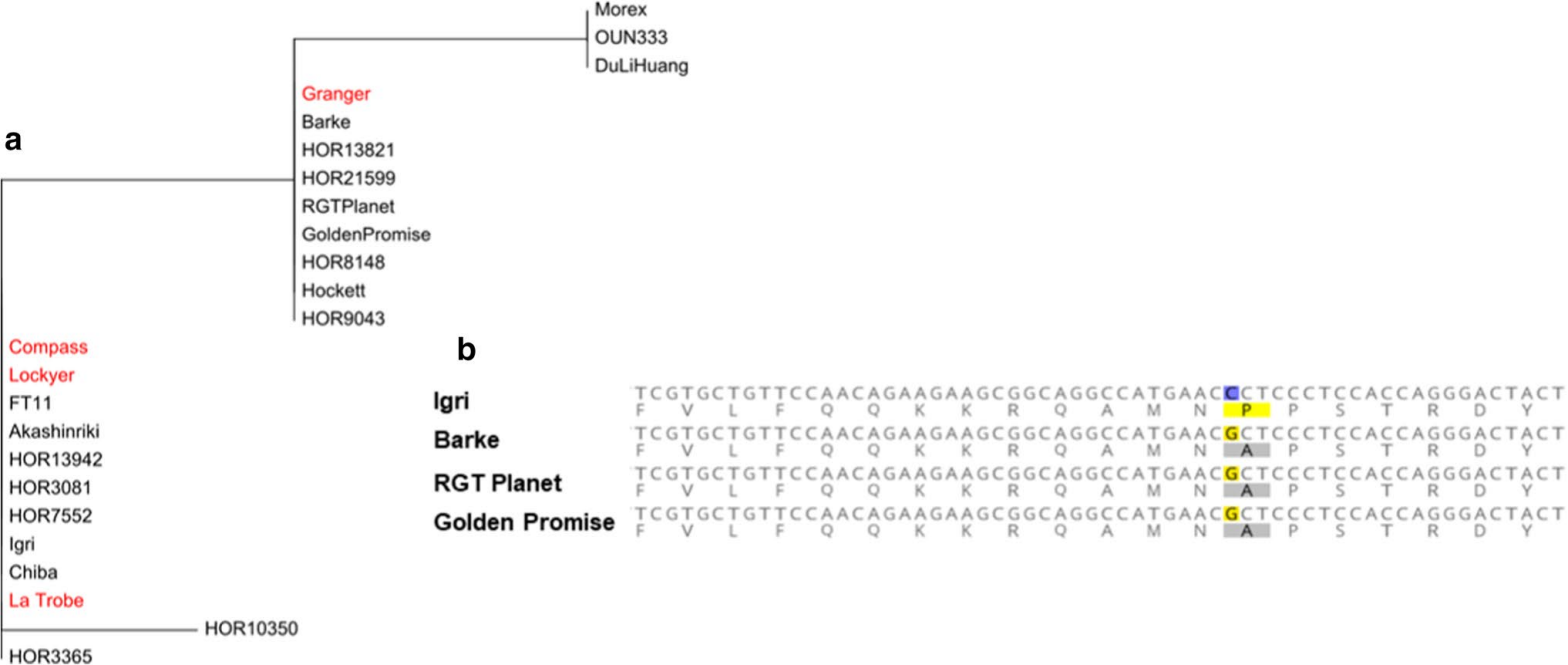
Sequence analysis of four parental cultivars Compass, GrangeR, La Trobe and Lockyer with 20 barley accessions from the pan-genome dataset. **a** Phylogenetic tree based on 13 genetic variants in the *CEN* gene region. **b** A C-to-G SNP caused an amino acid change from Proline to Alanine in cvs. Barke, RGT Planet, and Golden Promise

Analysis using 13 SNPs obtained from previous studies of Hill et al. (2019a, b) and the pan-genome sequences of 20 barley accessions (Jayakodi et al. 2020) in the *CENTRORADIALIS* (*CEN*) gene region suggested that the *CEN* sequence from cv. GrangeR is similar to many well-known commercial cultivars such as Barke, RGT Planet and Golden Promise (Fig. 5a). It is worth noting that cvs. GrangeR, Barke, RGT Planet and Golden Promise carrying a C-to-A SNP in the last exon, which caused a change from Proline to Alanine in the translated protein (Fig. 5b). It is highly possible that the GrangeR genotype, carrying a C-to-A SNP in the last exon of the *CEN* gene, is responsible for associating the linkage region on chromosome 2H with flowering time.

### The role of Ppd-H1 gene in photoperiod response

**Fig. 6:**
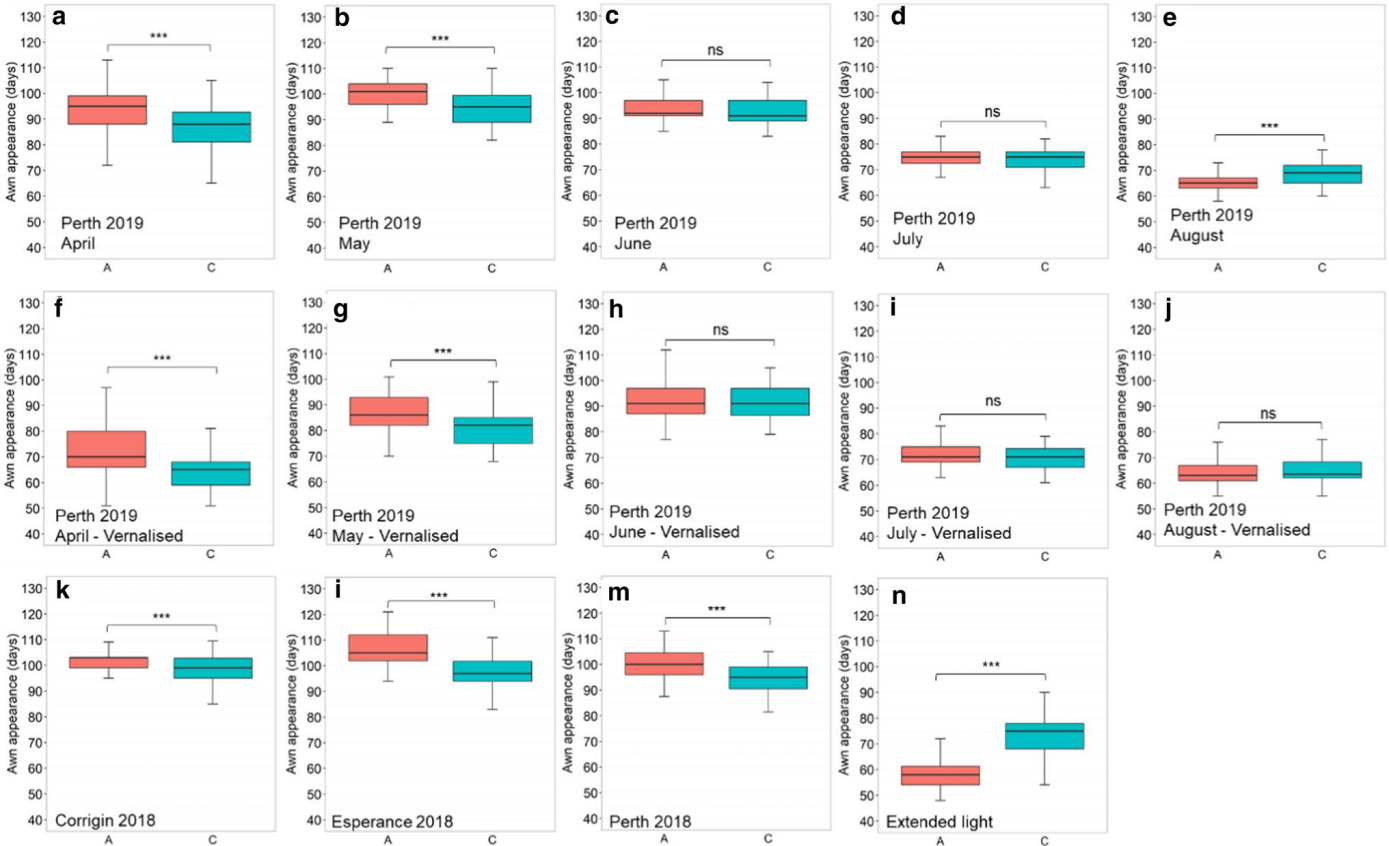
Boxplots for flowering time of different D2H29454480_AC QTN in different trials. **a**–**e** Non-vernalisation trials with different sowing times from April to August in Perth 2019. **f**–**j** Trials with vernalisation were sown from April to August in Perth 2019. **k**–**m** Trials at different locations in Corrigin, Esperance and Perth 2018. **n** Extended photoperiod trial. The population was divided into two groups according to allele types in each trial. The X-axis represents the two alleles, while the Y-axis corresponds to flowering time (Z49). Student’s t-test results shown for comparison: ns: not significant at *p*-value = 0.05, *** *p*-value < 0.001

The two genotypes of the D2H29454480_AC QTN, located near the *Ppd-H1* gene region, showed significant differences in flowering time in multiple trials, including vernalised/non-vernalised and various locations. The A genotype of D2H29454480_AC QTN was associated with later flowering time in Corrigin and Esperance and in some of the sowing dates in Perth (April and May) (Fig. 6 and Table S6). It is worth noting that the flowering time between the A genotype and C genotype of the D2H29454480_AC QTN showed no significant difference (at *p*-value = 0.05) when sowed later in June and July.

While exhibited later flowering habits when sowed in April and May, the A genotype D2H29454480_AC QTN promoted flowering in August sowing time in Perth (non-vernalised) and extended light trials (Fig. 6). This might suggest that different alleles of the D2H29454480_AC QTN are associated with different *Ppd-H1* alleles. However, plants following vernalisation treatment showed no significant difference in flowering time between the two genotype groups when sowed in August (Fig. 6e). Moreover, the C genotype of D2H29454480_AC QTN showed significantly earlier flowering time after vernalisation than the non-vernalised plants (Fig. 6f).

### Impact of the D2H638903073_TG QTN on vernalisation response

Four significant QTNs were detected by GWAS using flowering time variation data calculated from vernalised and non-vernalised trials (Fig. 3r–v and Table S4). These four QTNs can be divided into two groups, one group spanning approximately 8 Mb and located near the *Vrn-H1* gene region (D5H595203077_GA and D5H587440733_AG), the other group spanning around 400 kb on chromosome 2H (D2H638903073_TG and D2H639289577_GA).

Comparisons of flowering time between vernalised and non-vernalised plants suggested that different genotypes of the D2H638903073_TG QTN showed different responses to vernalisation. In trials conducted under normal conditions, plants carrying the T genotype of the D2H638903073_TG QTN only showed significant differences (at *p*-value = 0.05) in flowering time compared to the G genotype in the August sowing time (Table 2). However, the difference in flowering between the two genotype groups became more significant after vernalisation. Plants harbouring the T genotype of the D2H638903073_TG QTN exhibited significantly earlier flowering time than the G genotype in the vernalisation trials (Table 2). It is worth noting that the D2H638903073_TG QTN was not detected by GWAS using flowering time data in non-vernalised and vernalised trails (Fig. 3 and Table S4).

**Table 2:** Mean flowering time and standard error of different genotypes of the D2H638903073_TG QTN in different sowing times and vernalisation trials in Perth, 2019

Sowing time	Non-vernalised			Vernalised		
	Genotype		*p*-value	Genotype		*p*-value
	T	G		T	G	
April 2019	89.4 ± 0.8	92.5 ± 1.7	0.114	68.2 ± 0.8	72.1 ± 1.9	0.068
May 2019	97.5 ± 0.6	98.2 ± 1.2	0.582	83.2 ± 0.7	87.8 ± 1.6	0.011^*^
June 2019	92.4 ± 0.4	94.6 ± 1.3	0.105	90.9 ± 0.6	94.4 ± 1.3	0.033^*^
July 2019	74.3 ± 0.5	76.2 ± 0.9	0.136	70.3 ± 0.5	74.5 ± 0.7	< 0.001^***^
August 2019	65.7 ± 0.4	67.4 ± 0.7	0.038^*^	64.2 ± 0.5	67.5 ± 0.9	0.003^**^

^*^p-value < 0.05, **p-value < 0.01,***p-value < 0.001

## Discussion

### Identification of QTN for flowering time using multi-locus GWAS approaches in a MAGIC population

The identification of superior alleles is crucial for breeding of superior varieties in crops, including barley. In this study, we identified favourable alleles for flowering time by using the MAGIC population with Multi-locus GWAS approaches. Multiple QTNs associated with flowering time were detected, especially on chromosomes 2H, 3H, 5H and 7H (Fig. 3). It is worth noting that these significant QTNs are inherited from different parental cultivars, which suggested that these cultivars carried different phenology genes/alleles (Table 1 and Table S4).

### Multiple sowing times, different locations, vernalisation treatment and extended photoperiod provided insights of genes responsible for environmental response

The utilisation of different environmental conditions played an important role in the detection of QTNs that respond to specific conditions. One of the most important photoperiod pathway genes, the *Ppd-H1* gene, was previously characterised by Turner et al. (2005). Several studies of Acquaah (2012); Turner et al. (2005) and Wiegmann et al. (2019) reported that the photosensitive allele of the *Ppd-H1* gene promoted flowering under long-day conditions. However, the impacts of different alleles of the *Ppd-H1* gene on flowering under Australian growing conditions have not been fully understood. Our analysis based on previous studies by Hill et al. (2019a, b) showed that 342 out of 448 selected barley (76.3%) accessions selected from Europe and North America carried the G-to-T SNP in the CCT domain, which is characterised as the photoperiod insensitive allele (*ppd-H1*). However, only 121 out of 328 (36.9%) Australian barley accessions carried the *ppd-H1* allele. In this study, plants carrying the A genotype of D2H29454480_AC QTN promoted flowering in the extended photoperiod condition, which suggested that the A genotype is associated with the photoperiod sensitive allele (*Ppd-H1*), while the C genotype is associated with the photoperiod insensitive allele (*ppd-H1*). Analyses using different sowing times and locations indicated that the photoperiod sensitive allele (A genotype) exhibited later flowering habits when sowed in Esperance, Corrigin, April and May in Perth. The A genotype was only associated with earlier flowering for the August sowing time in Perth 2019 (Fig. 6), indicating that the photoperiod sensitive allele only promoted flowering in very late sowing times. Since barley in Australia is mostly sown from May to July under short-day conditions (11 h/day) compared to about 16 h/day of light in Europe (Pham et al. 2020), the *Ppd-H1* variant may be a better choice in Australian growing conditions, and delay flowering in frost susceptible area while accelerating flowering in higher latitude sites to avoid heat stress. Although there are a few studies that have reported the association of the *Ppd-H1* allele with late flowering habits (Bustos‐Korts et al. 2019; Merchuk-Ovnat et al. 2018; Ponce‐Molina et al. 2012; Saade et al. 2016), the underlying mechanism that caused such delay in flowering time in short-day conditions remains unclear. Borràs-Gelonch et al. (2011); Slafer and Rawson (1996) suggested that the photoperiod response of the *Ppd-H1* gene is further related to temperature.

Several stable regions detected by GWAS showed significant association with flowering time only in specific trials. For example, the significant QTN at the *PFT1* gene region (D5H430868101_AG) showed associations with flowering time in the Esperance and Corrigin trials in 2018 (Table 1). It is worth noting that these locations are very different in terms of temperature and rainfall. Corrigin has an annual rainfall of about 350 mm, which typically occurs from May to September while Esperance has about 600 mm of annual rainfall, mainly from May to December (Dang et al. 2020). Although the specific association of the *PFT1* gene with flowering time in barley is still unclear, studies in Arabidopsis suggested that the *PFT1* gene plays an essential role in response to light conditions (Cerdán and Chory 2003; Klose et al. 2012). *PFT1* regulates the expression of FLOWERING LOCUS T by acting as a downstream component of phytochrome A and B (phyA and phyB) (Cerdán and Chory 2003).

### CENTRORADIALIS shows stable associations with flowering time across various growing conditions

We were able to use the combination of GWAS results, high-quality SNPs from previous studies, and the pangenome sequences (Hill et al. 2019a, b; Jayakodi et al. 2020) to determine a specific mutation in the *CEN* gene region. Our result suggested that plants carrying a C-to-A SNP in the last exon of the *CEN* gene, inherited from cv. GrangeR, showed a delayed flowering habit compared to plants carrying Compass, La Trobe and Lockyer alleles. A previous study by Comadran et al. (2012) reported that the C-to-A SNP caused a change from Proline to Alanine in the translated protein and resulted in a delay in flowering time. Data obtained from different sowing times, various growing locations, vernalisation treatment and extended photoperiod trials confirmed that the impacts of *CEN* gene on flowering time were not affected by environmental conditions (Fig. 4 and Table S5). In our study, plants harbouring the *CEN* allele inherited from cv. GrangeR (A genotype of the D2H519658782_AC QTN) showed later flowering habits ranging from 3.7 to 12.2 days compared to the allele from cvs. Compass/La Trobe/Lockyer (Table S5). Previous studies reported that the Ala135 coding haplotype of the *CEN* gene (cv. GrangeR haplotype) was found to be better adapted to the cold and long growing season in Europe (Comadran et al. 2012, Fernandez-Calleja et al. 2021). Under these conditions, a prolonged growing period may help the plants utilise environmental resources for improving grain yield. The introduction of the cv. GrangeR Ala135 coding *CEN* haplotype to other Australian barley varieties could benefit Southern regions with longer raining seasons and later flowering required to escape frost damage.

### Novel combinations of genes associated with flowering from the recombinant population

The association of the semi-dwarf genes, *GA20ox2* and *DEP1*, with flowering enabled the utilisation of these genes in designing barley varieties with desired flowering time and height. Previous studies showed that the mutations in the *GA20ox2* region (*sdw1* gene) caused shorter plant height and delayed flowering time (Dang et al. 2020; Jia et al. 2009; Laurie et al. 1993; Xu et al. 2017). On the other hand, the *ari-e* gene (*DEP1* mutant) is widely used for breeding many successful Australian barley cultivars, including cvs. Hindmarsh and La Trobe, and causes shorter plant height with earlier flowering time (Dang et al. 2020).

Since genetic factors played an important role in controlling flowering time in the population (Table S7), introducing one alternative allele to the parental cultivar backgrounds could achieve new recombinant lines with later or earlier flowering time, and possibly improving grain yield. Different alleles of the *CEN* gene can be utilised for fine-tuning flowering time in plants carrying other phenology genes. For example, combining the *ari-e* allele from cv. La Trobe, which promotes flowering by up to 6 days (Dang et al. 2020), with the *CEN* allele from cv. GrangeR (A genotype), associated with later flowering, may result in shorter and later flowering plants compared to plants carrying only the *ari-e* allele. Such later flowering time can be utilised in designing varieties adapting to growing zones that are susceptible to frost damage. It is worth noting that some of the RILs showed later flowering habits of up to 7 days compared cv. Lockyer (the latest parental cultivar) when grown in the Esperance region, and earlier flowering habits of up to 21 days compared to cv. La Trobe when sown in August in Perth (Table S8).

In this study, we also obtained recombinant plants carrying the combinations of the photoperiod sensitive allele of the *Ppd-H1* gene (from cvs. La Trobe and Lockyer) and the later flowering genotype of the *CEN* gene (A genotype from cv. GrangeR). Moreover, these recombinant lines also carry different semi-dwarf genes (*sdw1*, *ari-e* or no semi-dwarf) and have various genetic backgrounds inherited from one of the parental cultivars (Table S9). The combination of the *sdw1* gene, the photoperiod sensitive allele *Ppd-H1* and the *CEN* allele from cv. GrangeR might result in barley plants with a very late flowering time. Such gene combinations can be utilised in the Southern high rainfall regions (such as Esperance) to take advantage of the longer growing period and minimise frost damage.

Cultivars La Trobe (developed in South Australia) and Lockyer (developed in Western Australia) carry different semi-dwarf alleles (*sdw1* in cv. Lockyer and *ari-e* in cv. La Trobe), but also the identical alleles at the four significant QTNs D2H29454480_AC (*Ppd-H1*), D2H519658782_AC (*CEN*), D5H430868101_AG (*PFT1*) and D2H638903073_TG. These similarities might be the keys to their success in Australian growing conditions, with La Trobe accounting for nearly 30% of barley grown in Western Australia. In this study, we created recombinant lines carrying the identical La Trobe and Lockyer alleles for these QTNs but with different semi-dwarf backgrounds (no semi-dwarf gene present or double-dwarf, *sdw1* + *ari-e*) (Table S9). The recombinant line with no semi-dwarf gene (MP0424) shared a similar genetic background with cv. Compass, while the double-dwarf recombinant line (MP0576) was closely related to cv. Lockyer (Fig. S2 and Table S9). Although double-dwarf plants may offer higher lodging resistance, they are less competitive with weeds and might not be ideal for growing sites experiencing high weed pressure (O’Donovan et al., 2000). However, this germplasm is useful for studying gene interactions among semi-dwarf and other genes.

## Supplementary Information

Below is the link to the electronic supplementary material.

Supplementary file1 (DOCX 789 kb)

Supplementary file2 (DOCX 106 kb)

Supplementary file3 (XLSX 76 kb)

## Data Availability

The datasets generated during and/or analysed during the current study are available from the corresponding author on reasonable request.
