# Supplementary material for: Multi-locus genome-wide association studies reveal novel alleles for flowering time under vernalisation and extended photoperiod in a barley MAGIC population
Source: Theor Appl Genet. 2022 Jul 25;135(9):3087–102. doi: 10.1007/s00122-022-04169-x (PMC9482607; doi:10.1007/s00122-022-04169-x)
Supplement: Supplementary file 1 — Supplementary file1 (DOCX 789 kb) [file 122_2022_4169_MOESM1_ESM.docx]

# Multi-locus genome-wide association studies reveal novel alleles for flowering time under vernalisation and extended photoperiod in a barley MAGIC population

**Viet Hoang Dang^1,2^, Camilla Beate Hill^1^, Xiao-Qi Zhang^1^, Tefera Tolera Angessa^1^, Lee-Anne McFawn^2^, Chengdao Li^1,2, 🖂^**

^1^ Western Crop Genetics Alliance, Agricultural Sciences, College of Science, Health, Engineering and Education, Murdoch University, Murdoch, Perth, WA, Australia

^2^ Department of Primary Industries and Regional Development, Perth, WA, Australia

🖂 Chengdao Li

[c.li@murdoch.edu.au](mailto:c.li@murdoch.edu.au)

**
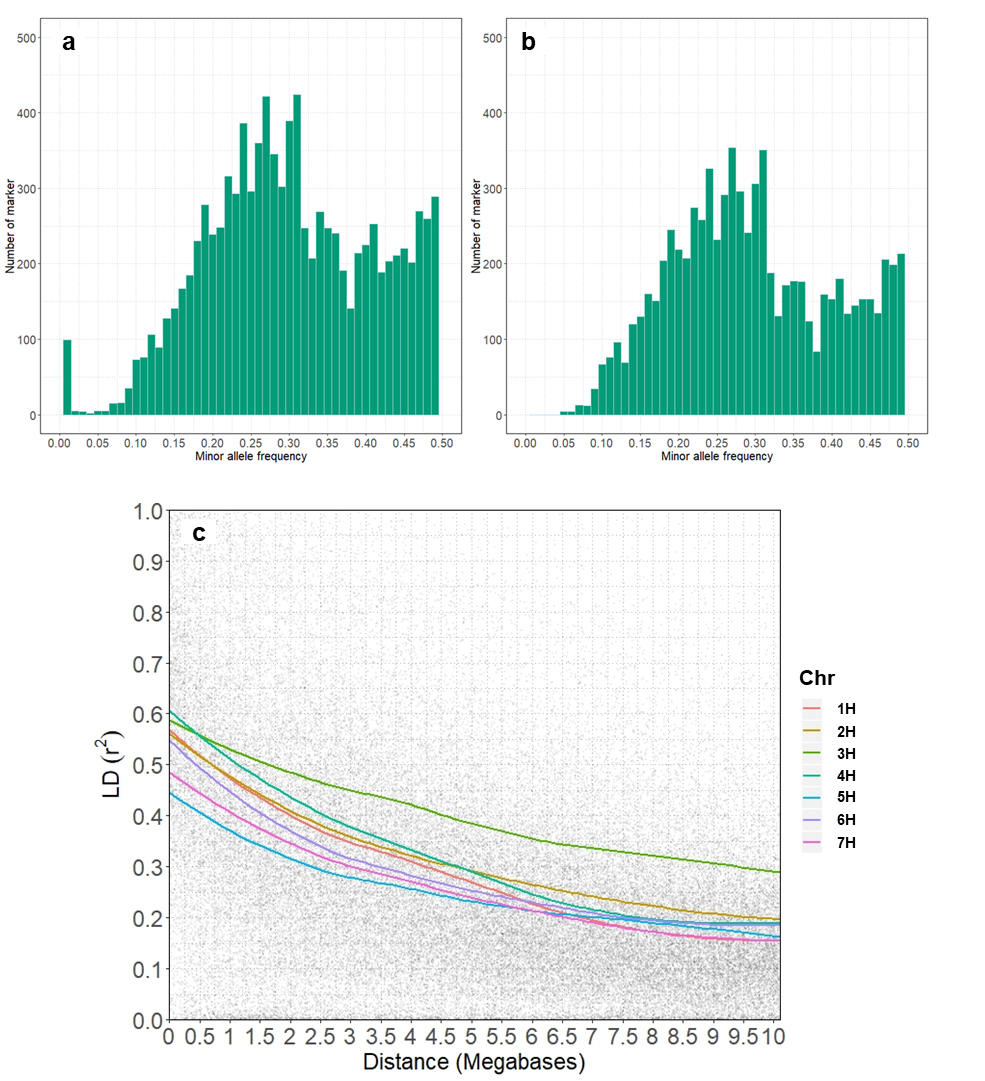
**

**Fig. S1** Minor allele frequency distribution of the (a) original marker set, and (b) the marker set after filtering with minor allele frequency threshold of 0.05, missingness threshold of 0.2 using PLINK 1.9 and imputed using BEAGLE 5.0; (c) linkage disequilibrium (LD, r^2^) of markers. The coloured lines depict second-degree LOESS curves for each chromosome


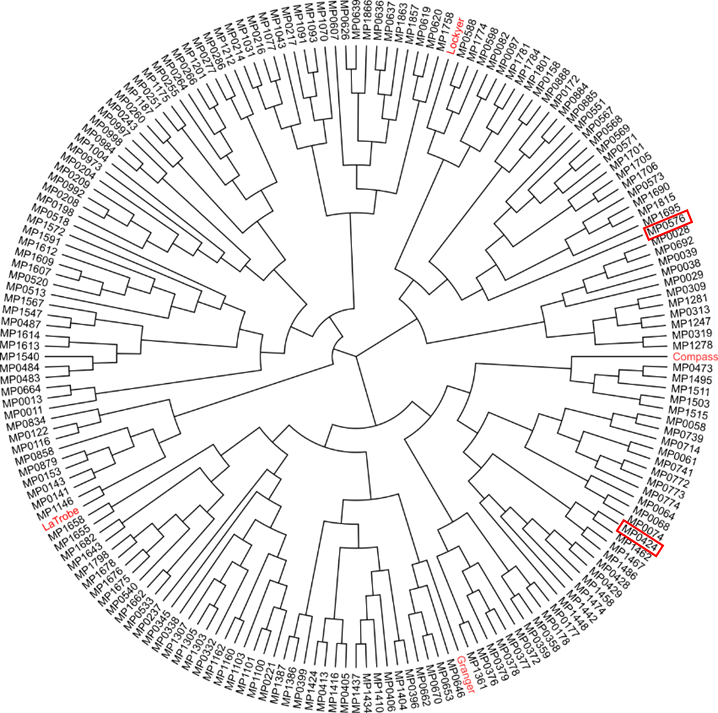


**Fig. S2** Phylogenetic tree constructed from 184 recombinant lines and four parental cultivars. Red boxes depicted recombinant lines carrying same major phenology QTNs with cvs. La Trobe and Lockyer but deferent semi-dwarf backgrounds.


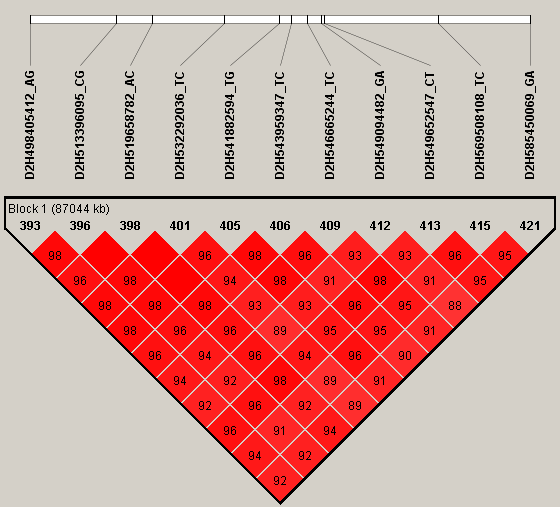


**Fig. S3** Linkage analysis result of 11 genetic markers associated with flowering time spanning a 100 Mb region on chromosome 2H
