# Supplementary material for: Multi-locus genome-wide association studies reveal novel alleles for flowering time under vernalisation and extended photoperiod in a barley MAGIC population
Source: Theor Appl Genet. 2022 Jul 25;135(9):3087–102. doi: 10.1007/s00122-022-04169-x (PMC9482607; doi:10.1007/s00122-022-04169-x)
Supplement: Supplementary file 2 — Supplementary file2 (DOCX 106 kb) [file 122_2022_4169_MOESM2_ESM.docx]

# Multi-locus genome-wide association studies reveal novel alleles for flowering time under vernalisation and extended photoperiod in a barley MAGIC population

**Viet Hoang Dang^1,2^, Camilla Beate Hill^1^, Xiao-Qi Zhang^1^, Tefera Tolera Angessa^1^, Lee-Anne McFawn^2^, Chengdao Li^1,2, 🖂^**

^1^ Western Crop Genetics Alliance, Agricultural Sciences, College of Science, Health, Engineering and Education, Murdoch University, Murdoch, Perth, WA, Australia

^2^ Department of Primary Industries and Regional Development, Perth, WA, Australia

🖂 Chengdao Li

[c.li@murdoch.edu.au](mailto:c.li@murdoch.edu.au)**Table S1** List of trials conducted in Perth, Corrigin and Esperance, Western Australia with different sowing dates, vernalisation and extended photoperiod from 2017 to 2019

| **Trial** | **Sowing date** | **Location** | **Treatment** |
| --- | --- | --- | --- |
| 1 | 30^th^ May 2017 | Perth | No |
| 2 | 31^st^ May 2018 | Corrigin | No |
| 3 | 09^th^ May 2018 | Esperance | No |
| 4 | 24^th^ May 2018 | Perth | No |
| 5 | 18^th^ April 2019 | Perth | No |
| 6 | 15^th^ May 2019 | Perth | No |
| 7 | 18^th^ June 2019 | Perth | No |
| 8 | 18^th^ July 2019 | Perth | No |
| 9 | 06^th^ August 2019 | Perth | No |
| 10 | 15^th^ May 2019 | Perth | 18-hour photoperiod |
| 11 | 18^th^ April 2019 | Perth | 6-week vernalisation |
| 12 | 15^th^ May 2019 | Perth | 6-week vernalisation |
| 13 | 18^th^ June 2019 | Perth | 6-week vernalisation |
| 14 | 18^th^ July 2019 | Perth | 6-week vernalisation |
| 15 | 06^th^ August 2019 | Perth | 4-week vernalisation |

**Table S2** Additional KASP markers for phenology and semi-dwarf genes

| **Chromosome** | **Position** | **Gene name** |
| --- | --- | --- |
| 1H | 26,220,293 |  |
| 1H | 411,257,151 | *ZCCTc* |
| 1H | 458,874,152 | *STK* |
| 1H | 490,940,854 | *GA20ox2-2* |
| 1H | 490,941,064 | *GA20ox2-2* |
| 1H | 497,763,802 | *LCC* |
| 1H | 514,098,322 | *FT3 (PPD-H2)* |
| 1H | 515,519,668 | *GA2ox4* |
| 1H | 538,165,591 | *GA2ox1* |
| 1H | 556,900,431 | *ELF3* |
| 1H | 556,903,491 | *ELF3* |
| 1H | 558,225,979 | *CMF6b* |
| 1H | 558,226,159 | *CMF6b* |
| 2H | 2,087,259 | *EFS* |
| 2H | 2,087,380 | *EFS* |
| 2H | 2,097,742 | *EFS* |
| 2H | 6,177,044 | *LIM3* |
| 2H | 6,177,615 | *LIM3* |
| 2H | 29,123,942 | *Ppd-H1* |
| 2H | 29,125,815 | *Ppd-H1* |
| 2H | 40,288,295 | *CDF1* |
| 2H | 68,835,904 | *FT4* |
| 2H | 128,268,917 | *GW7* |
| 2H | 128,269,079 | *GW7* |
| 2H | 128,271,153 | *GW7* |
| 2H | 201,118,546 | *CBF8A* |
| 2H | 201,118,611 | *CBF8A* |
| 2H | 432,062,326 | *BM8* |
| 2H | 432,062,544 | *BM8* |
| 2H | 611,559,546 | *FTL5* |
| 2H | 611,564,428 |  |
| 2H | 673,850,122 | *FD* |
| 2H | 673,851,856 | *FD* |
| 2H | 685,787,135 | *AGL32* |
| 2H | 685,788,746 | *AGL32* |
| 2H | 685,788,836 | *AGL32* |
| 2H | 697,166,570 | *LFY1* |
| 2H | 697,168,237 | *LFY1* |
| 2H | 742,246,169 | *GA3ox1* |
| 2H | 742,246,651 | *GA3ox1* |
| 2H | 742,248,143 | *GA3ox1* |
| 3H | 3,244,232 | *ELF7* |
| 3H | 83,331,503 | *GA3ox2* |
| 3H | 119,252,315 | *FT2* |
| 3H | 548,754,394 | *GA2ox3* |
| 3H | 548,755,241 | *GA2ox3* |
| 3H | 630,547,167 | *GA20ox3* |
| 3H | 634,079,978 | *GA20ox2* |
| 3H | 634,081,121 | *GA20ox2* |
| 3H | 634,081,556 | *GA20ox2* |
| 3H | 634,932,524 | *CIGARP-2* |
| 3H | 634,933,639 | *CIGARP-2* |
| 3H | 634,933,651 | *CIGARP-2* |
| 3H | 699,527,121 | *GA2betadiox7* |
| 3H | 699,527,655 | *GA2betadiox7* |
| 3H | 699,527,991 | *GA2betadiox7* |
| 4H | 6,132,648 | *GRP7a* |
| 4H | 16,669,060 | *SLN1* |
| 4H | 16,670,109 | *SLN1* |
| 4H | 16,671,691 | *SLN1* |
| 4H | 60,195,252 | *GID1L2-8* |
| 4H | 60,195,395 | *GID1L2-8* |
| 4H | 300,701,680 | *FKF1* |
| 4H | 467,567,098 | *BB* |
| 4H | 581,616,973 | *GARMP* |
| 4H | 627,080,516 | *CMF4* |
| 4H | 627,080,614 | *CMF4* |
| 4H | 645,064,549 | *FT5* |
| 4H | 645,065,497 | *FT5* |
| 5H | 31,837,959 | *PIF4* |
| 5H | 482,215,793 | *DEP1* |
| 5H | 533,361,846 |  |
| 5H | 559,462,552 | *CBF9* |
| 5H | 559,462,683 | *CBF9* |
| 5H | 559,462,764 | *CBF9* |
| 5H | 559,673,257 | *CBF4A* |
| 5H | 559,673,372 | *CBF4A* |
| 5H | 559,673,567 | *CBF4A* |
| 5H | 559,687,817 |  |
| 5H | 559,687,961 | *CBF2A* |
| 5H | 559,688,580 | *CBF2A* |
| 5H | 560,196,083 | *CBF14* |
| 5H | 560,196,134 | *CBF14* |
| 5H | 560,196,229 | *CBF14* |
| 5H | 560,570,288 | *CBF3* |
| 5H | 560,571,061 | *CBF3* |
| 5H | 560,571,115 | *CBF3* |
| 5H | 560,571,147 | *CBF3* |
| 5H | 560,586,997 | *CBF10A* |
| 5H | 560,588,142 | *CBF10A* |
| 5H | 560,588,191 | *CBF10A* |
| 5H | 560,588,206 | *CBF10A* |
| 5H | 560,588,256 |  |
| 5H | 560,732,097 | *CBF6* |
| 5H | 560,732,713 | *CBF6* |
| 5H | 565,157,545 | *PRR95* |
| 5H | 598,231,895 | *ADA2* |
| 5H | 598,564,939 | *PhyC* |
| 5H | 599,123,281 | *BM5 (Vrn-H1)* |
| 5H | 599,332,514 | *AGLG1* |
| 5H | 599,332,735 | *AGLG1* |
| 6H | 6,262,431 | *AGL1* |
| 6H | 6,264,731 | *AGL1* |
| 6H | 6,268,303 | *AGL1* |
| 6H | 70,578,602 | *ZTLb* |
| 6H | 70,582,296 | *ZTLb* |
| 6H | 72,969,182 | *VEL1* |
| 6H | 139,882,589 | *TT16* |
| 6H | 139,884,132 | *TT16* |
| 6H | 374,868,207 | *TOC1* |
| 6H | 374,868,982 | *TOC1* |
| 6H | 384,632,464 | *Cry2* |
| 6H | 384,633,137 | *Cry2* |
| 6H | 504,463,075 | *CO2* |
| 6H | 504,465,056 | *CO2* |
| 7H | 37,608,103 | *MADS25-2* |
| 7H | 37,619,514 | *MADS25-2* |
| 7H | 37,904,213 | *MADS25-3* |
| 7H | 37,905,096 | *MADS25-3* |
| 7H | 37,905,206 | *MADS25-3* |
| 7H | 49,217,139 | *CO8* |
| 7H | 49,217,216 | *CO8* |
| Un | 12,158,311 | *BM3* |

**Table S3** Mean and standard error of flowering time (Z49) of different trials in Perth in 2019.

| **Trial** | **Treatment** | **Z49** | **n** |
| --- | --- | --- | --- |
| April 2019 | No | 90.2 ± 0.7 | 185 |
|  | Vernalisation | 69.3 ± 0.7 | 188 |
| May 2019 | No | 97.6 ± 0.5 | 185 |
|  | Vernalisation | 84.3 ± 0.6 | 188 |
|  | Extended day length | 63.8 ± 0.7 | 187 |
| June 2019 | No | 93 ± 0.4 | 188 |
|  | Vernalisation | 91.9 ± 0.6 | 159 |
| July 2019 | No | 74.8 ± 0.5 | 137 |
|  | Vernalisation | 71.4 ± 0.5 | 115 |
| August 2019 | No | 66.1 ± 0.3 | 187 |
|  | Vernalisation | 64.9 ± 0.5 | 166 |

**Table S4** Significant quantitative trait nucleotides (QTNs) from Genome-wide association studies for flowering time

| **Trial** | **Method** | **Marker** | **Chr** | **Pos** | **QTN Effect** | **LOD** | **Compass** | **GrangeR** | **La Trobe** | **Lockyer** |
| --- | --- | --- | --- | --- | --- | --- | --- | --- | --- | --- |
| Perth 2017 | mrMLM | D2H28394307_TC | 2 | 28394307 | 1.6 | 5.84 | T | T | C | C |
| Perth 2017 | mrMLM | D2H549652547_CT | 2 | 549652547 | -2.84 | 10.32 | C | T | C | C |
| Perth 2017 | mrMLM | D3H497561170_GA | 3 | 497561170 | -1.59 | 3.38 | G | A | G | G |
| Perth 2017 | mrMLM | D3H631832650_CG | 3 | 631832650 | -2.12 | 8.03 | C | G | C | G |
| Perth 2017 | FASTmrMLM | D2H28394307_TC | 2 | 28394307 | 1.41 | 5.84 | T | T | C | C |
| Perth 2017 | FASTmrMLM | D2H549652547_CT | 2 | 549652547 | -2.65 | 10.32 | C | T | C | C |
| Perth 2017 | FASTmrMLM | D3H497561170_GA | 3 | 497561170 | -1.21 | 3.38 | G | A | G | G |
| Perth 2017 | FASTmrMLM | D3H631832650_CG | 3 | 631832650 | -2.04 | 8.03 | C | G | C | G |
| Perth 2017 | FASTmrEMMA | D2H28394307_TC | 2 | 28394307 | 3.23 | 5.71 | T | T | C | C |
| Perth 2017 | FASTmrEMMA | D3H635379323_GC | 3 | 635379323 | 4.9 | 9.88 | G | C | G | C |
| Perth 2017 | pLARmEB | D2H28394307_TC | 2 | 28394307 | 1.39 | 6.49 | T | T | C | C |
| Perth 2017 | pLARmEB | D2H549652547_CT | 2 | 549652547 | -2.43 | 9.84 | C | T | C | C |
| Perth 2017 | pKWmEB | D1H489138501_GC | 1 | 489138501 | 0.81 | 3.69 | C | G | G | G |
| Perth 2017 | pKWmEB | D2H549652547_CT | 2 | 549652547 | -1.63 | 6.03 | C | T | C | C |
| Perth 2017 | pKWmEB | D3H631680288_GC | 3 | 631680288 | -1.49 | 4.74 | C | G | C | G |
| Perth 2017 | ISIS EM-BLASSO | D2H31462699_TG | 2 | 31462699 | 1.45 | 5.96 | T | T | G | G |
| Perth 2017 | ISIS EM-BLASSO | D2H549652547_CT | 2 | 549652547 | -2.55 | 9.5 | C | T | C | C |
| Perth 2017 | ISIS EM-BLASSO | D3H631832650_CG | 3 | 631832650 | -2.43 | 12.18 | C | G | C | G |
| Perth 2019 June Vernalised | mrMLM | D2H525245905_AC | 2 | 525245905 | -3.77 | 5.78 | A | C | A | A |
| Perth 2019 June Vernalised | mrMLM | D3H677376626_CT | 3 | 677376626 | -2.95 | 3.23 | C | C | T | C |
| Perth 2019 June Vernalised | FASTmrEMMA | D3H606013063_CG | 3 | 606013063 | -3.98 | 3 | C | G | G | G |
| Perth 2019 June Vernalised | FASTmrEMMA | D7H118564441_AT | 7 | 118564441 | 4.96 | 4.44 | T | A | A | T |
| Perth 2019 June Vernalised | pLARmEB | D2H513396095_CG | 2 | 513396095 | -4.12 | 8.63 | C | G | C | C |
| Perth 2019 June Vernalised | pLARmEB | D5H478394957_AG | 5 | 478394957 | -2.3 | 3.74 | G | G | A | G |
| Perth 2019 June Vernalised | pLARmEB | D7H32776273_GA | 7 | 32776273 | 1.77 | 3.08 | A | G | A | G |
| Perth 2019 June Vernalised | pKWmEB | D2H513396095_CG | 2 | 513396095 | -2.82 | 5.14 | C | G | C | C |
| Perth 2019 June Vernalised | ISIS EM-BLASSO | D2H549094482_GA | 2 | 549094482 | -3.69 | 8.3 | A | G | A | A |
| Perth 2019 June Vernalised | ISIS EM-BLASSO | D3H677376626_CT | 3 | 677376626 | -2.31 | 3.79 | C | C | T | C |
| Perth 2019 June Vernalised | ISIS EM-BLASSO | D5H478394957_AG | 5 | 478394957 | -2.27 | 4.15 | G | G | A | G |
| Perth 2019 June Vernalised | ISIS EM-BLASSO | D7H32776273_GA | 7 | 32776273 | 2 | 3.85 | A | G | A | G |
| Perth 2019 July Vernalised | FASTmrEMMA | D2H20093178_TC | 2 | 20093178 | -4.49 | 3.56 | C | T | T | C |
| Perth 2019 July Vernalised | pLARmEB | K1H538165591_GA | 1 | 538165591 | 1.09 | 3.15 | G | G | A | A |
| Perth 2019 July Vernalised | pLARmEB | D1H548125741_CT | 1 | 548125741 | -1.92 | 3.39 | C | C | T | C |
| Perth 2019 July Vernalised | pLARmEB | D2H271995988_TC | 2 | 271995988 | 2.35 | 6.42 | T | C | T | T |
| Perth 2019 July Vernalised | pLARmEB | D5H617105681_AT | 5 | 617105681 | -1.22 | 4.6 | A | A | T | T |
| Perth 2019 July Vernalised | pLARmEB | D5H651511097_CG | 5 | 651511097 | 1.43 | 4.29 | C | C | G | C |
| Perth 2019 July Vernalised | pLARmEB | D7H625140060_AG | 7 | 625140060 | 2.06 | 3.46 | G | A | G | G |
| Perth 2019 July Vernalised | ISIS EM-BLASSO | K1H538165591_GA | 1 | 538165591 | 1.45 | 3.77 | G | G | A | A |
| Perth 2019 July Vernalised | ISIS EM-BLASSO | D7H625140060_AG | 7 | 625140060 | 2.5 | 3.68 | G | A | G | G |
| Perth 2019 August Vernalised | mrMLM | D1H1421695_AC | 1 | 1421695 | 2.5 | 3.26 | C | A | C | C |
| Perth 2019 August Vernalised | mrMLM | D1H554606691_AC | 1 | 554606691 | -2.12 | 3.33 | C | A | A | A |
| Perth 2019 August Vernalised | mrMLM | D2H37222451_AG | 2 | 37222451 | -1.94 | 3.71 | G | G | A | A |
| Perth 2019 August Vernalised | mrMLM | D5H600471239_AC | 5 | 600471239 | -2.69 | 3.36 | C | A | A | A |
| Perth 2019 August Vernalised | FASTmrMLM | D2H37222451_AG | 2 | 37222451 | -1.48 | 3.56 | G | G | A | A |
| Perth 2019 August Vernalised | FASTmrEMMA | D2H432062575_GA | 2 | 432062575 | 4.41 | 3.48 | G | A | G | G |
| Perth 2019 August Vernalised | pLARmEB | D2H37222451_AG | 2 | 37222451 | -1.24 | 3.27 | G | G | A | A |
| Perth 2019 August Vernalised | ISIS EM-BLASSO | D2H432062575_GA | 2 | 432062575 | 2.36 | 4.34 | G | A | G | G |
| Perth 2019 August Vernalised | ISIS EM-BLASSO | D5H390501082_TG | 5 | 390501082 | -2.26 | 3.26 | T | T | G | T |
| Esperance 2018 | mrMLM | D1H4653106_AG | 1 | 4653106 | 1.72 | 3.1 | G | A | G | A |
| Esperance 2018 | mrMLM | D2H541882594_TG | 2 | 541882594 | -2.98 | 5.59 | G | T | G | G |
| Esperance 2018 | mrMLM | D2H29454480_AC | 2 | 29454480 | 4.85 | 19.71 | C | C | A | A |
| Esperance 2018 | mrMLM | D3H631832727_GC | 3 | 631832727 | 3.88 | 13.41 | G | C | G | C |
| Esperance 2018 | mrMLM | D7H37802896_AG | 7 | 37802896 | 3.29 | 9.53 | A | G | A | G |
| Esperance 2018 | FASTmrMLM | D1H4653106_AG | 1 | 4653106 | 2.01 | 5.62 | G | A | G | A |
| Esperance 2018 | FASTmrMLM | D2H29454480_AC | 2 | 29454480 | 4.71 | 17.32 | C | C | A | A |
| Esperance 2018 | FASTmrMLM | D3H631680288_GC | 3 | 631680288 | -3.93 | 13.8 | C | G | C | G |
| Esperance 2018 | FASTmrMLM | D7H40095600_TC | 7 | 40095600 | -3.26 | 9.16 | T | C | T | C |
| Esperance 2018 | FASTmrEMMA | D1H4653106_AG | 1 | 4653106 | 3.01 | 3.5 | G | A | G | A |
| Esperance 2018 | FASTmrEMMA | D2H29564269_TC | 2 | 29564269 | -9.29 | 18.46 | C | C | T | T |
| Esperance 2018 | FASTmrEMMA | D2H585450069_GA | 2 | 585450069 | 4.76 | 4.51 | G | A | G | G |
| Esperance 2018 | FASTmrEMMA | D3H631832727_GC | 3 | 631832727 | 7.45 | 12.77 | G | C | G | C |
| Esperance 2018 | FASTmrEMMA | D7H40095600_TC | 7 | 40095600 | -5.89 | 8.35 | T | C | T | C |
| Esperance 2018 | pLARmEB | D1H4653106_AG | 1 | 4653106 | 1.55 | 4.82 | G | A | G | A |
| Esperance 2018 | pLARmEB | D2H29454480_AC | 2 | 29454480 | 4.58 | 22.85 | C | C | A | A |
| Esperance 2018 | pLARmEB | D2H541882594_TG | 2 | 541882594 | -2.84 | 5.93 | G | T | G | G |
| Esperance 2018 | pLARmEB | D3H631832727_GC | 3 | 631832727 | 3.81 | 17.68 | G | C | G | C |
| Esperance 2018 | pLARmEB | D5H430868101_AG | 5 | 430868101 | -2.5 | 4.49 | G | A | A | A |
| Esperance 2018 | pLARmEB | K5H559462552_AG | 5 | 559462552 | 1.13 | 3.38 | G | A | A | A |
| Esperance 2018 | pLARmEB | D5H597950604_TC | 5 | 597950604 | 0 | 3.56 | T | C | T | T |
| Esperance 2018 | pLARmEB | D7H40095600_TC | 7 | 40095600 | -2.64 | 8.42 | T | C | T | C |
| Esperance 2018 | pKWmEB | D1H4653106_AG | 1 | 4653106 | 1.86 | 6.43 | G | A | G | A |
| Esperance 2018 | pKWmEB | D2H29454480_AC | 2 | 29454480 | 5 | 19.35 | C | C | A | A |
| Esperance 2018 | pKWmEB | D2H38574929_AG | 2 | 38574929 | -2.07 | 4.78 | G | A | A | A |
| Esperance 2018 | pKWmEB | D2H271999272_GA | 2 | 271999272 | 1.91 | 4.04 | G | A | G | G |
| Esperance 2018 | pKWmEB | D3H631680288_GC | 3 | 631680288 | -2.08 | 6.14 | C | G | C | G |
| Esperance 2018 | pKWmEB | D4H510209595_GA | 4 | 510209595 | -1.95 | 5.38 | G | G | G | A |
| Esperance 2018 | pKWmEB | D5H587440727_CT | 5 | 587440727 | 1.37 | 3.82 | C | T | C | C |
| Esperance 2018 | pKWmEB | D6H6216705_CT | 6 | 6216705 | -1.35 | 3.95 | T | T | C | T |
| Esperance 2018 | ISIS EM-BLASSO | D1H4653106_AG | 1 | 4653106 | 1.66 | 5.56 | G | A | G | A |
| Esperance 2018 | ISIS EM-BLASSO | D2H29454480_AC | 2 | 29454480 | 4.71 | 25.11 | C | C | A | A |
| Esperance 2018 | ISIS EM-BLASSO | D3H631832727_GC | 3 | 631832727 | 3.76 | 17.43 | G | C | G | C |
| Esperance 2018 | ISIS EM-BLASSO | D5H45772858_TC | 5 | 45772858 | -1.27 | 3.52 | T | T | C | T |
| Esperance 2018 | ISIS EM-BLASSO | K5H559462552_AG | 5 | 559462552 | 1.14 | 3.87 | G | A | A | A |
| Esperance 2018 | ISIS EM-BLASSO | D5H590274735_GA | 5 | 590274735 | 1.81 | 4.85 | A | G | G | G |
| Esperance 2018 | ISIS EM-BLASSO | D5H597800435_CT | 5 | 597800435 | 1.45 | 4.36 | C | T | C | C |
| Esperance 2018 | ISIS EM-BLASSO | D7H37802896_AG | 7 | 37802896 | 2.55 | 6.86 | A | G | A | G |
| Corrigin 2018 | mrMLM | D1H4653106_AG | 1 | 4653106 | 1.62 | 4.8 | G | A | G | A |
| Corrigin 2018 | mrMLM | D2H29454480_AC | 2 | 29454480 | 1.74 | 4.3 | C | C | A | A |
| Corrigin 2018 | mrMLM | D3H631832727_GC | 3 | 631832727 | 3.3 | 10.71 | G | C | G | C |
| Corrigin 2018 | mrMLM | D7H37802896_AG | 7 | 37802896 | 2.42 | 6.15 | A | G | A | G |
| Corrigin 2018 | FASTmrMLM | D1H4653106_AG | 1 | 4653106 | 1.44 | 4.57 | G | A | G | A |
| Corrigin 2018 | FASTmrMLM | D1H5762961_GA | 1 | 5762961 | 0.72 | 3.01 | G | G | A | A |
| Corrigin 2018 | FASTmrMLM | D2H29454480_AC | 2 | 29454480 | 1.51 | 4.56 | C | C | A | A |
| Corrigin 2018 | FASTmrMLM | D3H631832650_CG | 3 | 631832650 | -2.89 | 10.86 | C | G | C | G |
| Corrigin 2018 | FASTmrMLM | D7H37802896_AG | 7 | 37802896 | 2.05 | 6.11 | A | G | A | G |
| Corrigin 2018 | FASTmrEMMA | D3H634804408_CA | 3 | 634804408 | 5.1 | 5.85 | C | C | C | A |
| Corrigin 2018 | pLARmEB | D1H4653106_AG | 1 | 4653106 | 1.28 | 3.99 | G | A | G | A |
| Corrigin 2018 | pLARmEB | D2H29454480_AC | 2 | 29454480 | 1.57 | 5.72 | C | C | A | A |
| Corrigin 2018 | pLARmEB | D2H57616692_GC | 2 | 57616692 | 0.96 | 3.08 | G | C | C | C |
| Corrigin 2018 | pLARmEB | D3H631832727_GC | 3 | 631832727 | 2.82 | 11.88 | G | C | G | C |
| Corrigin 2018 | pLARmEB | D5H430868101_AG | 5 | 430868101 | -1.42 | 3.18 | G | A | A | A |
| Corrigin 2018 | pLARmEB | D7H37802896_AG | 7 | 37802896 | 1.87 | 5.63 | A | G | A | G |
| Corrigin 2018 | ISIS EM-BLASSO | D2H29454480_AC | 2 | 29454480 | 1.84 | 6.96 | C | C | A | A |
| Corrigin 2018 | ISIS EM-BLASSO | D2H57616692_GC | 2 | 57616692 | 1.25 | 4.24 | G | C | C | C |
| Corrigin 2018 | ISIS EM-BLASSO | D2H543959347_TC | 2 | 543959347 | 1.32 | 3.42 | T | C | T | T |
| Corrigin 2018 | ISIS EM-BLASSO | D3H631832650_CG | 3 | 631832650 | -2.94 | 10.69 | C | G | C | G |
| Corrigin 2018 | ISIS EM-BLASSO | D5H430868101_AG | 5 | 430868101 | -1.94 | 4.72 | G | A | A | A |
| Corrigin 2018 | ISIS EM-BLASSO | D7H37802896_AG | 7 | 37802896 | 1.71 | 4.72 | A | G | A | G |
| Perth 2019 Extended photoperiod | mrMLM | D2H38635094_TC | 2 | 38635094 | 5.04 | 3.5 | C | C | T | T |
| Perth 2019 Extended photoperiod | FASTmrMLM | D2H38635094_TC | 2 | 38635094 | 2.31 | 3.4 | C | C | T | T |
| Perth 2019 Extended photoperiod | FASTmrEMMA | D2H38635094_TC | 2 | 38635094 | 5.31 | 3.53 | C | C | T | T |
| Perth 2019 Extended photoperiod | pLARmEB | D2H38635094_TC | 2 | 38635094 | 2.31 | 3.4 | C | C | T | T |
| Perth 2019 Extended photoperiod | pKWmEB | K2H29123942_AG | 2 | 29123942 | 4.26 | 9.5 | A | A | G | G |
| Perth 2019 Extended photoperiod | pKWmEB | D2H38635094_TC | 2 | 38635094 | 2.1 | 3.5 | C | C | T | T |
| Perth 2019 Extended photoperiod | ISIS EM-BLASSO | D2H38635094_TC | 2 | 38635094 | 2.32 | 3.53 | C | C | T | T |
| Vernalised/Non-Vernalised April | FASTmrMLM | D5H595203077_GA | 5 | 595203077 | -1.92 | 3.96 | G | A | G | G |
| Vernalised/Non-Vernalised April | FASTmrEMMA | D5H595203077_GA | 5 | 595203077 | -3.49 | 3.35 | G | A | G | G |
| Vernalised/Non-Vernalised April | pLARmEB | D5H595203077_GA | 5 | 595203077 | -1.92 | 3.92 | G | A | G | G |
| Vernalised/Non-Vernalised April | pKWmEB | D5H595203077_GA | 5 | 595203077 | -1.44 | 3.14 | G | A | G | G |
| Vernalised/Non-Vernalised April | ISIS EM-BLASSO | D5H587440733_AG | 5 | 587440733 | 1.62 | 3.61 | A | G | A | A |
| Vernalised/Non-Vernalised May | FASTmrEMMA | D2H638903073_TG | 2 | 638903073 | -3.76 | 3.24 | T | G | T | T |
| Vernalised/Non-Vernalised May | ISIS EM-BLASSO | D2H639289577_GA | 2 | 639289577 | 2.02 | 3.54 | A | G | A | A |
| Perth 2018 | mrMLM | D2H29454480_AC | 2 | 29454480 | 3.29 | 14.83 | C | C | A | A |
| Perth 2018 | mrMLM | D2H543959347_TC | 2 | 543959347 | 3.7 | 12.44 | T | C | T | T |
| Perth 2018 | mrMLM | D3H631832650_CG | 3 | 631832650 | -3.07 | 12.69 | C | G | C | G |
| Perth 2018 | mrMLM | K5H482215793_IndelC_CA | 5 | 482215793 | -2.23 | 3.98 | C | C | A | C |
| Perth 2018 | FASTmrMLM | D2H29454480_AC | 2 | 29454480 | 3.11 | 15.44 | C | C | A | A |
| Perth 2018 | FASTmrMLM | D2H543959347_TC | 2 | 543959347 | 3.5 | 12.05 | T | C | T | T |
| Perth 2018 | FASTmrMLM | D3H631832650_CG | 3 | 631832650 | -2.95 | 11.87 | C | G | C | G |
| Perth 2018 | FASTmrMLM | K5H482215793_IndelC_CA | 5 | 482215793 | -1.73 | 3.93 | C | C | A | C |
| Perth 2018 | FASTmrEMMA | D2H29564269_TC | 2 | 29564269 | -5.66 | 12.37 | C | C | T | T |
| Perth 2018 | FASTmrEMMA | D2H543959347_TC | 2 | 543959347 | 6.5 | 10.45 | T | C | T | T |
| Perth 2018 | pLARmEB | D2H29454480_AC | 2 | 29454480 | 2.85 | 13.72 | C | C | A | A |
| Perth 2018 | pLARmEB | D2H408967031_AG | 2 | 408967031 | -3.32 | 10.62 | A | G | A | A |
| Perth 2018 | pLARmEB | D2H748648671_AG | 2 | 748648671 | -1 | 3.73 | A | A | A | G |
| Perth 2018 | pLARmEB | D3H631812217_AG | 3 | 631812217 | 3 | 13.49 | G | A | G | A |
| Perth 2018 | pLARmEB | D7H425661183_CT | 7 | 425661183 | 1.29 | 3.96 | T | C | T | C |
| Perth 2018 | pKWmEB | D2H29564269_TC | 2 | 29564269 | -2.51 | 8.15 | C | C | T | T |
| Perth 2018 | pKWmEB | D2H532292036_TC | 2 | 532292036 | 2.27 | 6.93 | T | C | T | T |
| Perth 2018 | pKWmEB | D3H631832650_CG | 3 | 631832650 | -2.17 | 9.02 | C | G | C | G |
| Perth 2018 | ISIS EM-BLASSO | D2H29454480_AC | 2 | 29454480 | 2.87 | 14.19 | C | C | A | A |
| Perth 2018 | ISIS EM-BLASSO | D2H546665244_TC | 2 | 546665244 | 3.17 | 11.71 | T | C | T | T |
| Perth 2018 | ISIS EM-BLASSO | D3H631832650_CG | 3 | 631832650 | -3.03 | 14.72 | C | G | C | G |
| Perth 2018 | ISIS EM-BLASSO | D4H473895443_AC | 4 | 473895443 | 1.56 | 4.59 | A | C | C | C |
| Perth 2018 | ISIS EM-BLASSO | D5H39817622_AG | 5 | 39817622 | -1.25 | 3.15 | G | G | A | G |
| Perth 2018 | ISIS EM-BLASSO | D5H628711504_GA | 5 | 628711504 | -1.19 | 4.18 | A | A | G | A |
| Perth April 2019 | mrMLM | D2H549094482_GA | 2 | 549094482 | -5.19 | 9.47 | A | G | A | A |
| Perth April 2019 | mrMLM | D2H31462699_TG | 2 | 31462699 | 4.06 | 9.65 | T | T | G | G |
| Perth April 2019 | mrMLM | K2H685788836_CG | 2 | 685788836 | 3.14 | 3.92 | C | G | G | C |
| Perth April 2019 | mrMLM | D5H478394957_AG | 5 | 478394957 | -4.48 | 6.22 | G | G | A | G |
| Perth April 2019 | mrMLM | D7H32776273_GA | 7 | 32776273 | 4.85 | 8.39 | A | G | A | G |
| Perth April 2019 | FASTmrMLM | D2H31462699_TG | 2 | 31462699 | 3.72 | 11.17 | T | T | G | G |
| Perth April 2019 | FASTmrMLM | D2H549094482_GA | 2 | 549094482 | -4.84 | 8.97 | A | G | A | A |
| Perth April 2019 | FASTmrMLM | K2H685788836_CG | 2 | 685788836 | 2.51 | 3.62 | C | G | G | C |
| Perth April 2019 | FASTmrMLM | D5H478394957_AG | 5 | 478394957 | -3.92 | 6.25 | G | G | A | G |
| Perth April 2019 | FASTmrMLM | D7H32776273_GA | 7 | 32776273 | 4.42 | 8.33 | A | G | A | G |
| Perth April 2019 | FASTmrEMMA | D2H498405412_AG | 2 | 498405412 | 9.48 | 9.22 | G | A | G | G |
| Perth April 2019 | FASTmrEMMA | K3H634079978_GA | 3 | 634079978 | 5.53 | 4.82 | G | G | G | A |
| Perth April 2019 | FASTmrEMMA | D5H478394957_AG | 5 | 478394957 | -9.41 | 8.21 | G | G | A | G |
| Perth April 2019 | FASTmrEMMA | D7H32776273_GA | 7 | 32776273 | 7.82 | 6.9 | A | G | A | G |
| Perth April 2019 | pLARmEB | D2H31462699_TG | 2 | 31462699 | 3.44 | 10.57 | T | T | G | G |
| Perth April 2019 | pLARmEB | D2H549094482_GA | 2 | 549094482 | -5.07 | 10.63 | A | G | A | A |
| Perth April 2019 | pLARmEB | K3H634079978_GA | 3 | 634079978 | 3.06 | 6.05 | G | G | G | A |
| Perth April 2019 | pLARmEB | D5H478394957_AG | 5 | 478394957 | -4.57 | 8.89 | G | G | A | G |
| Perth April 2019 | pLARmEB | D5H587440733_AG | 5 | 587440733 | 1.59 | 3.61 | A | G | A | A |
| Perth April 2019 | pLARmEB | D7H32776273_GA | 7 | 32776273 | 3.99 | 7.18 | A | G | A | G |
| Perth April 2019 | pKWmEB | D2H29454480_AC | 2 | 29454480 | 3.27 | 6.87 | C | C | A | A |
| Perth April 2019 | pKWmEB | D2H402416491_AC | 2 | 402416491 | -3.26 | 5.22 | A | C | A | A |
| Perth April 2019 | pKWmEB | D5H478394957_AG | 5 | 478394957 | -3.51 | 5.76 | G | G | A | G |
| Perth April 2019 | ISIS EM-BLASSO | D2H29454480_AC | 2 | 29454480 | 3.71 | 9.41 | C | C | A | A |
| Perth April 2019 | ISIS EM-BLASSO | D2H549094482_GA | 2 | 549094482 | -5.16 | 12.3 | A | G | A | A |
| Perth April 2019 | ISIS EM-BLASSO | K3H634079978_GA | 3 | 634079978 | 2.81 | 5.38 | G | G | G | A |
| Perth April 2019 | ISIS EM-BLASSO | D5H478394957_AG | 5 | 478394957 | -4.71 | 9.47 | G | G | A | G |
| Perth April 2019 | ISIS EM-BLASSO | D5H587440733_AG | 5 | 587440733 | 1.52 | 3.18 | A | G | A | A |
| Perth April 2019 | ISIS EM-BLASSO | D7H32776273_GA | 7 | 32776273 | 3.77 | 6.56 | A | G | A | G |
| Perth May 2019 | mrMLM | D2H29454480_AC | 2 | 29454480 | 3.16 | 9.3 | C | C | A | A |
| Perth May 2019 | mrMLM | D3H634804408_CA | 3 | 634804408 | 2.48 | 5.53 | C | C | C | A |
| Perth May 2019 | mrMLM | K5H482215793_IndelC_CA | 5 | 482215793 | -3.33 | 5.84 | C | C | A | C |
| Perth May 2019 | mrMLM | D7H40095600_TC | 7 | 40095600 | -2.11 | 3.77 | T | C | T | C |
| Perth May 2019 | FASTmrMLM | D2H29454480_AC | 2 | 29454480 | 2.9 | 9.31 | C | C | A | A |
| Perth May 2019 | FASTmrMLM | D3H634804408_CA | 3 | 634804408 | 2.13 | 5.53 | C | C | C | A |
| Perth May 2019 | FASTmrMLM | K5H482215793_IndelC_CA | 5 | 482215793 | -2.83 | 5.84 | C | C | A | C |
| Perth May 2019 | FASTmrMLM | D7H40095600_TC | 7 | 40095600 | -1.65 | 3.78 | T | C | T | C |
| Perth May 2019 | FASTmrEMMA | D2H29564269_TC | 2 | 29564269 | -5.52 | 8.84 | C | C | T | T |
| Perth May 2019 | FASTmrEMMA | D3H634804408_CA | 3 | 634804408 | 4.34 | 5.55 | C | C | C | A |
| Perth May 2019 | FASTmrEMMA | D7H40095600_TC | 7 | 40095600 | -3.4 | 3.94 | T | C | T | C |
| Perth May 2019 | pLARmEB | D2H29454480_AC | 2 | 29454480 | 3.02 | 12.4 | C | C | A | A |
| Perth May 2019 | pLARmEB | D2H549094482_GA | 2 | 549094482 | -2.28 | 5.33 | A | G | A | A |
| Perth May 2019 | pLARmEB | D3H79426134_GT | 3 | 79426134 | -1.72 | 3.58 | T | G | T | T |
| Perth May 2019 | pLARmEB | D3H634804408_CA | 3 | 634804408 | 1.97 | 5.96 | C | C | C | A |
| Perth May 2019 | pLARmEB | K5H482215793_IndelC_CA | 5 | 482215793 | -3.08 | 8.07 | C | C | A | C |
| Perth May 2019 | pLARmEB | D7H111614504_TC | 7 | 111614504 | -0.92 | 3.04 | C | T | T | T |
| Perth May 2019 | pKWmEB | D2H29454480_AC | 2 | 29454480 | 2.35 | 6.69 | C | C | A | A |
| Perth May 2019 | pKWmEB | D2H592062371_GA | 2 | 592062371 | 0.98 | 3.15 | G | A | G | A |
| Perth May 2019 | pKWmEB | D5H382294832_AG | 5 | 382294832 | -2.31 | 3.67 | G | G | A | G |
| Perth May 2019 | ISIS EM-BLASSO | D2H29454480_AC | 2 | 29454480 | 3.02 | 13.15 | C | C | A | A |
| Perth May 2019 | ISIS EM-BLASSO | D2H546665244_TC | 2 | 546665244 | 2.56 | 7.03 | T | C | T | T |
| Perth May 2019 | ISIS EM-BLASSO | D3H634804408_CA | 3 | 634804408 | 2.06 | 6.77 | C | C | C | A |
| Perth May 2019 | ISIS EM-BLASSO | K5H482215793_IndelC_CA | 5 | 482215793 | -2.18 | 3.13 | C | C | A | C |
| Perth May 2019 | ISIS EM-BLASSO | D7H111614504_TC | 7 | 111614504 | -1.02 | 3.48 | C | T | T | T |
| Per June 2019 | mrMLM | D2H519658782_AC | 2 | 519658782 | 3.99 | 17.34 | C | A | C | C |
| Per June 2019 | mrMLM | D3H631680288_GC | 3 | 631680288 | -1.61 | 4.93 | C | G | C | G |
| Per June 2019 | mrMLM | K5H482215793_IndelC_CA | 5 | 482215793 | -1.84 | 3.57 | C | C | A | C |
| Per June 2019 | mrMLM | D7H37802896_AG | 7 | 37802896 | 2.03 | 6.92 | A | G | A | G |
| Per June 2019 | FASTmrMLM | D2H519658782_AC | 2 | 519658782 | 3.99 | 17.34 | C | A | C | C |
| Per June 2019 | FASTmrMLM | D3H631680288_GC | 3 | 631680288 | 0 | 4.93 | C | G | C | G |
| Per June 2019 | FASTmrMLM | K5H482215793_IndelC_CA | 5 | 482215793 | -1.58 | 3.57 | C | C | A | C |
| Per June 2019 | FASTmrMLM | D7H37802896_AG | 7 | 37802896 | 1.55 | 6.92 | A | G | A | G |
| Per June 2019 | FASTmrEMMA | D2H519658782_AC | 2 | 519658782 | 7.95 | 18.29 | C | A | C | C |
| Per June 2019 | FASTmrEMMA | D3H634581648_GA | 3 | 634581648 | 3.13 | 5.18 | G | G | G | A |
| Per June 2019 | FASTmrEMMA | K5H482215793_IndelC_CA | 5 | 482215793 | -3.14 | 4.02 | C | C | A | C |
| Per June 2019 | FASTmrEMMA | D7H37802896_AG | 7 | 37802896 | 3.24 | 5.88 | A | G | A | G |
| Per June 2019 | pLARmEB | D3H631680288_GC | 3 | 631680288 | -1.37 | 5.07 | C | G | C | G |
| Per June 2019 | pLARmEB | K5H482215793_IndelC_CA | 5 | 482215793 | -1.43 | 3.64 | C | C | A | C |
| Per June 2019 | pLARmEB | D7H37802896_AG | 7 | 37802896 | 1.81 | 6.85 | A | G | A | G |
| Per June 2019 | pKWmEB | D2H408967031_AG | 2 | 408967031 | -3.13 | 11.37 | A | G | A | A |
| Per June 2019 | ISIS EM-BLASSO | D2H519658782_AC | 2 | 519658782 | 3.87 | 16.59 | C | A | C | C |
| Per June 2019 | ISIS EM-BLASSO | D3H631680288_GC | 3 | 631680288 | -1.37 | 4.95 | C | G | C | G |
| Per June 2019 | ISIS EM-BLASSO | K5H482215793_IndelC_CA | 5 | 482215793 | -1.43 | 3.73 | C | C | A | C |
| Per June 2019 | ISIS EM-BLASSO | D7H37802896_AG | 7 | 37802896 | 1.81 | 7 | A | G | A | G |
| Perth July 2019 | mrMLM | K5H482215793_IndelC_CA | 5 | 482215793 | -4.23 | 5.9 | C | C | A | C |
| Perth July 2019 | mrMLM | D7H20324931_GA | 7 | 20324931 | 3.24 | 5.23 | A | G | A | G |
| Perth July 2019 | FASTmrMLM | K5H482215793_IndelC_CA | 5 | 482215793 | -3.86 | 3.3 | C | C | A | C |
| Perth July 2019 | FASTmrMLM | D7H20324931_GA | 7 | 20324931 | 2.74 | 5.43 | A | G | A | G |
| Perth July 2019 | FASTmrEMMA | D7H20324931_GA | 7 | 20324931 | 5.46 | 5.69 | A | G | A | G |
| Perth July 2019 | pLARmEB | D2H513396095_CG | 2 | 513396095 | -1.78 | 3.34 | C | G | C | C |
| Perth July 2019 | pLARmEB | K5H482215793_IndelC_CA | 5 | 482215793 | -2.97 | 4.22 | C | C | A | C |
| Perth July 2019 | pLARmEB | D7H20324931_GA | 7 | 20324931 | 2.53 | 4.74 | A | G | A | G |
| Perth July 2019 | ISIS EM-BLASSO | D2H128357515_AT | 2 | 128357515 | 1.68 | 3.23 | T | A | T | T |
| Perth July 2019 | ISIS EM-BLASSO | D5H42145240_AG | 5 | 42145240 | 2.63 | 3.28 | A | A | G | A |
| Perth July 2019 | ISIS EM-BLASSO | K5H482215793_IndelC_CA | 5 | 482215793 | -2.79 | 4.06 | C | C | A | C |
| Perth July 2019 | ISIS EM-BLASSO | D7H20324931_GA | 7 | 20324931 | 2.59 | 4.89 | A | G | A | G |
| Perth August 2019 | mrMLM | K2H29123942_AG | 2 | 29123942 | 2.02 | 11.05 | A | A | G | G |
| Perth August 2019 | mrMLM | D2H569508108_TC | 2 | 569508108 | 2.35 | 9.36 | T | C | T | T |
| Perth August 2019 | mrMLM | D4H580004566_AG | 4 | 580004566 | 2.44 | 4.93 | A | A | A | G |
| Perth August 2019 | FASTmrMLM | K2H29123942_AG | 2 | 29123942 | 1.61 | 7.68 | A | A | G | G |
| Perth August 2019 | FASTmrMLM | D2H569508108_TC | 2 | 569508108 | 1.77 | 6.65 | T | C | T | T |
| Perth August 2019 | FASTmrMLM | D6H511517814_AG | 6 | 511517814 | 1.05 | 3.98 | A | A | A | G |
| Perth August 2019 | FASTmrMLM | D7H37802896_AG | 7 | 37802896 | 1.03 | 4.29 | A | G | A | G |
| Perth August 2019 | FASTmrEMMA | K2H29123942_AG | 2 | 29123942 | 3.29 | 7.95 | A | A | G | G |
| Perth August 2019 | FASTmrEMMA | D2H569508108_TC | 2 | 569508108 | 3.57 | 6.72 | T | C | T | T |
| Perth August 2019 | FASTmrEMMA | D6H506826032_CT | 6 | 506826032 | -2.05 | 3.74 | T | T | T | C |
| Perth August 2019 | FASTmrEMMA | D7H37802896_AG | 7 | 37802896 | 1.95 | 4.01 | A | G | A | G |
| Perth August 2019 | pLARmEB | K2H29123942_AG | 2 | 29123942 | 1.41 | 6.67 | A | A | G | G |
| Perth August 2019 | pLARmEB | D2H402416491_AC | 2 | 402416491 | -1.5 | 5.41 | A | C | A | A |
| Perth August 2019 | pLARmEB | D5H579459306_CA | 5 | 579459306 | 0.96 | 3.69 | C | A | C | C |
| Perth August 2019 | pLARmEB | D6H511517814_AG | 6 | 511517814 | 1.27 | 6.05 | A | A | A | G |
| Perth August 2019 | pLARmEB | D7H37802896_AG | 7 | 37802896 | 1.01 | 3.94 | A | G | A | G |
| Perth August 2019 | pLARmEB | D7H601732139_CG | 7 | 601732139 | 0.82 | 4.35 | C | C | G | G |
| Perth August 2019 | pLARmEB | D7H635235854_CG | 7 | 635235854 | 0.9 | 4.47 | G | G | C | C |
| Perth August 2019 | pKWmEB | K2H29123942_AG | 2 | 29123942 | 1.67 | 8.94 | A | A | G | G |
| Perth August 2019 | pKWmEB | D4H578442430_CT | 4 | 578442430 | 1.41 | 6.79 | C | C | C | T |
| Perth August 2019 | pKWmEB | D6H516794731_TC | 6 | 516794731 | 0 | 4.87 | T | C | C | T |
| Perth August 2019 | pKWmEB | D6H22764172_TC | 6 | 22764172 | 0 | 3.06 | T | C | T | T |
| Perth August 2019 | ISIS EM-BLASSO | K2H29123942_AG | 2 | 29123942 | 1.84 | 12.55 | A | A | G | G |
| Perth August 2019 | ISIS EM-BLASSO | D2H569508108_TC | 2 | 569508108 | 1.96 | 8.48 | T | C | T | T |
| Perth August 2019 | ISIS EM-BLASSO | D4H578442430_CT | 4 | 578442430 | 1.63 | 3.74 | C | C | C | T |
| Perth April 2019 Vernalised | mrMLM | D2H31462699_TG | 2 | 31462699 | 3.22 | 5.57 | T | T | G | G |
| Perth April 2019 Vernalised | mrMLM | D2H513396095_CG | 2 | 513396095 | -5.1 | 9.26 | C | G | C | C |
| Perth April 2019 Vernalised | mrMLM | D3H631832727_GC | 3 | 631832727 | 3.59 | 9.93 | G | C | G | C |
| Perth April 2019 Vernalised | mrMLM | D6H5210171_TA | 6 | 5210171 | 3.77 | 5.01 | T | A | T | A |
| Perth April 2019 Vernalised | mrMLM | D7H40095600_TC | 7 | 40095600 | -3.94 | 7.98 | T | C | T | C |
| Perth April 2019 Vernalised | FASTmrMLM | D2H31462699_TG | 2 | 31462699 | 2.54 | 5.73 | T | T | G | G |
| Perth April 2019 Vernalised | FASTmrMLM | D2H498405412_AG | 2 | 498405412 | 4.89 | 11.15 | G | A | G | G |
| Perth April 2019 Vernalised | FASTmrMLM | D3H631832727_GC | 3 | 631832727 | 4 | 11.32 | G | C | G | C |
| Perth April 2019 Vernalised | FASTmrMLM | D5H562614128_GC | 5 | 562614128 | 2.51 | 3.61 | C | G | C | C |
| Perth April 2019 Vernalised | FASTmrMLM | D6H5210171_TA | 6 | 5210171 | 3.34 | 5.2 | T | A | T | A |
| Perth April 2019 Vernalised | FASTmrMLM | D7H40095600_TC | 7 | 40095600 | -3.69 | 8.5 | T | C | T | C |
| Perth April 2019 Vernalised | FASTmrEMMA | D2H498405412_AG | 2 | 498405412 | 10.09 | 10.07 | G | A | G | G |
| Perth April 2019 Vernalised | FASTmrEMMA | D3H631832727_GC | 3 | 631832727 | 6.44 | 6.79 | G | C | G | C |
| Perth April 2019 Vernalised | FASTmrEMMA | D6H5210171_TA | 6 | 5210171 | 5.56 | 3.85 | T | A | T | A |
| Perth April 2019 Vernalised | FASTmrEMMA | D7H40095600_TC | 7 | 40095600 | -7.47 | 7.75 | T | C | T | C |
| Perth April 2019 Vernalised | pLARmEB | D2H31462699_TG | 2 | 31462699 | 2.68 | 6.93 | T | T | G | G |
| Perth April 2019 Vernalised | pLARmEB | D2H513396095_CG | 2 | 513396095 | -5.32 | 13.66 | C | G | C | C |
| Perth April 2019 Vernalised | pLARmEB | D3H631832727_GC | 3 | 631832727 | 3.75 | 10.57 | G | C | G | C |
| Perth April 2019 Vernalised | pLARmEB | D4H502678478_CT | 4 | 502678478 | 2.22 | 4.58 | C | T | T | T |
| Perth April 2019 Vernalised | pLARmEB | D5H562614128_GC | 5 | 562614128 | 2.96 | 4.79 | C | G | C | C |
| Perth April 2019 Vernalised | pLARmEB | D6H5210171_TA | 6 | 5210171 | 3.5 | 5.96 | T | A | T | A |
| Perth April 2019 Vernalised | pLARmEB | D7H40095600_TC | 7 | 40095600 | -2.97 | 5.81 | T | C | T | C |
| Perth April 2019 Vernalised | pKWmEB | D2H29454480_AC | 2 | 29454480 | 2.77 | 4.98 | C | C | A | A |
| Perth April 2019 Vernalised | pKWmEB | D2H498405412_AG | 2 | 498405412 | 4.21 | 9.35 | G | A | G | G |
| Perth April 2019 Vernalised | pKWmEB | D3H631832727_GC | 3 | 631832727 | 2.28 | 4.76 | G | C | G | C |
| Perth April 2019 Vernalised | pKWmEB | D7H40095600_TC | 7 | 40095600 | -2.42 | 4.93 | T | C | T | C |
| Perth April 2019 Vernalised | ISIS EM-BLASSO | D1H108240893_TG | 1 | 108240893 | -1.33 | 3.03 | G | G | T | T |
| Perth April 2019 Vernalised | ISIS EM-BLASSO | D2H31462699_TG | 2 | 31462699 | 2.65 | 6.14 | T | T | G | G |
| Perth April 2019 Vernalised | ISIS EM-BLASSO | D2H453687559_TC | 2 | 453687559 | -4.77 | 10.07 | C | T | C | C |
| Perth April 2019 Vernalised | ISIS EM-BLASSO | D3H631832727_GC | 3 | 631832727 | 3.91 | 10.85 | G | C | G | C |
| Perth April 2019 Vernalised | ISIS EM-BLASSO | D5H562614128_GC | 5 | 562614128 | 2.46 | 3.62 | C | G | C | C |
| Perth April 2019 Vernalised | ISIS EM-BLASSO | D6H5210171_TA | 6 | 5210171 | 3.63 | 6.4 | T | A | T | A |
| Perth April 2019 Vernalised | ISIS EM-BLASSO | D7H40095600_TC | 7 | 40095600 | -3.33 | 6.92 | T | C | T | C |
| Perth May 2019 Vernalised | mrMLM | D2H31462699_TG | 2 | 31462699 | 3.36 | 6.7 | T | T | G | G |
| Perth May 2019 Vernalised | mrMLM | D2H696974995_GA | 2 | 696974995 | 2.92 | 3.02 | G | A | G | G |
| Perth May 2019 Vernalised | mrMLM | D3H631680288_GC | 3 | 631680288 | -3.16 | 5.16 | C | G | C | G |
| Perth May 2019 Vernalised | FASTmrMLM | D2H31462699_TG | 2 | 31462699 | 2.9 | 6.7 | T | T | G | G |
| Perth May 2019 Vernalised | FASTmrMLM | D2H696974995_GA | 2 | 696974995 | 2.09 | 3.02 | G | A | G | G |
| Perth May 2019 Vernalised | FASTmrMLM | D3H631680288_GC | 3 | 631680288 | -2.79 | 5.16 | C | G | C | G |
| Perth May 2019 Vernalised | FASTmrEMMA | D2H28501872_CT | 2 | 28501872 | 5.03 | 4.74 | T | T | C | C |
| Perth May 2019 Vernalised | FASTmrEMMA | D2H585450069_GA | 2 | 585450069 | 6.48 | 5.05 | G | A | G | G |
| Perth May 2019 Vernalised | pLARmEB | D2H28501872_CT | 2 | 28501872 | 2.23 | 4.09 | T | T | C | C |
| Perth May 2019 Vernalised | pLARmEB | D2H569508108_TC | 2 | 569508108 | 2.65 | 3.83 | T | C | T | T |
| Perth May 2019 Vernalised | pLARmEB | D3H631680288_GC | 3 | 631680288 | -2.69 | 5.73 | C | G | C | G |
| Perth May 2019 Vernalised | pLARmEB | D7H30983729_GA | 7 | 30983729 | 2.49 | 4.08 | A | G | A | G |
| Perth May 2019 Vernalised | pKWmEB | D2H547422026_CA | 2 | 547422026 | -2.19 | 4.75 | A | C | A | A |
| Perth May 2019 Vernalised | pKWmEB | D2H29564269_TC | 2 | 29564269 | -2.45 | 4.41 | C | C | T | T |
| Perth May 2019 Vernalised | pKWmEB | D3H631680288_GC | 3 | 631680288 | -1.69 | 3.78 | C | G | C | G |
| Perth May 2019 Vernalised | ISIS EM-BLASSO | D2H29564269_TC | 2 | 29564269 | -2.83 | 6.65 | C | C | T | T |
| Perth May 2019 Vernalised | ISIS EM-BLASSO | D2H570296260_TC | 2 | 570296260 | -2.87 | 4.8 | C | T | C | C |
| Perth May 2019 Vernalised | ISIS EM-BLASSO | D2H703934445_CT | 2 | 703934445 | -1.87 | 3.16 | C | T | C | C |
| Perth May 2019 Vernalised | ISIS EM-BLASSO | D3H631680288_GC | 3 | 631680288 | -2.82 | 6.22 | C | G | C | G |
| Perth May 2019 Vernalised | ISIS EM-BLASSO | D7H30983729_GA | 7 | 30983729 | 2.54 | 4.29 | A | G | A | G |

**Table S5** Mean and standard error of flowering time (Z49) of different genotypes of the D2H519658782_AC QTN in different trials

| **Location** | **Sowing time** | **Treatment** | **Genotype** | |
| --- | --- | --- | --- | --- |
|  |  |  | **A** | **C** |
| Corrigin | May 2018 | No | 102.8 ± 0.7 | 99.1 ± 0.4 |
| Esperance | May 2018 | No | 108.6 ± 1.5 | 101.3 ± 0.6 |
| Perth | May 2017 | No | 104.5 ± 0.7 | 98.6 ± 0.4 |
|  | May 2018 | No | 103.8 ± 0.9 | 96.3 ± 0.5 |
|  | April 2019 | No | 98.4 ± 1.8 | 88.4 ± 0.8 |
|  |  | Vernalisation | 79.3 ± 2.0 | 67.1 ± 0.7 |
|  | May 2019 | No | 101.6 ± 1.2 | 96.6 ± 0.5 |
|  |  | Vernalisation | 91.5 ± 1.5 | 82.8 ± 0.7 |
|  |  | Extended photoperiod | 69.4 ± 1.8 | 62.7 ± 0.8 |
|  | June 2019 | No | 100.2 ± 0.9 | 91.3 ± 0.4 |
|  |  | Vernalisation | 100.1 ± 1.1 | 90.1 ± 0.5 |
|  | July 2019 | No | 78.3 ± 0.9 | 73.9 ± 0.5 |
|  |  | Vernalisation | 76 ± 0.8 | 70.4 ± 0.4 |
|  | August 2019 | No | 69.3 ± 0.7 | 65.4 ± 0.3 |
|  |  | Vernalisation | 68.9 ± 1.0 | 64.1 ± 0.5 |

**Table S6** Mean and standard error of flowering time (Z49) of different genotypes of the D2H29454480_AC QTN in different trials

| **Location** | **Sowing time** | **Treatment** | **Genotype** | |
| --- | --- | --- | --- | --- |
|  |  |  | **A** | **C** |
| Corrigin | May 2018 | No | 101.8 ± 0.4 | 98.2 ± 0.8 |
| Esperance | May 2018 | No | 106.4 ± 0.7 | 97.0 ± 1.0 |
| Perth | May 2017 | No | 101.2 ± 0.5 | 98.4 ± 0.8 |
|  | May 2018 | No | 100.1 ± 0.6 | 94.7 ± 0.8 |
|  | April 2019 | No | 93.9 ± 0.9 | 86.0 ± 1.2 |
|  |  | Vernalisation | 72.9 ± 1.0 | 65.0 ± 1.0 |
|  | May 2019 | No | 100.2 ± 0.6 | 94.6 ± 0.8 |
|  |  | Vernalisation | 87.1 ± 0.7 | 81.1 ± 1.2 |
|  |  | Extended photoperiod | 58.6 ± 0.6 | 72.9 ± 1.2 |
|  | June 2019 | No | 93.6 ± 0.5 | 92.7 ± 0.6 |
|  |  | Vernalisation | 92.1 ± 0.8 | 92.0 ± 1.0 |
|  | July 2019 | No | 74.9 ± 0.6 | 74.7 ± 0.9 |
|  |  | Vernalisation | 71.9 ± 0.6 | 70.5 ± 0.6 |
|  | August 2019 | No | 65.0 ± 0.4 | 68.5 ± 0.6 |
|  |  | Vernalisation | 64.7 ± 0.6 | 65.8 ± 0.8 |
